# Supplementary material for: Individualised screening for diabetic retinopathy: the ISDR study—rationale, design and methodology for a randomised controlled trial comparing annual and individualised risk-based variable-interval screening
Source: BMJ Open. 2019 Jun 17;9(6):e025788. doi: 10.1136/bmjopen-2018-025788 (PMC6588999; doi:10.1136/bmjopen-2018-025788)
Supplement: Author's manuscript [file bmjopen-2018-025788.draft_revisions.pdf]

BMJ Open is committed to open peer review. As part of this commitment we make the peer review history of every article we publish publicly available.

When an article is published we post the peer reviewers' comments and the authors' responses online. We also post the versions of the paper that were used during peer review. These are the versions that the peer review comments apply to.

The versions of the paper that follow are the versions that were submitted during the peer review process. They are not the versions of record or the final published versions. They should not be cited or distributed as the published version of this manuscript.

BMJ Open is an open access journal and the full, final, typeset and author-corrected version of record of the manuscript is available on our site with no access controls, subscription charges or pay-per-view fees (http://bmjopen.bmj.com).

If you have any questions on BMJ Open's open peer review process please email info.bmjopen@bmj.com

BMJ Open

Individualised Screening for Diabetic Retinopathy: the ISDR study. Rationale, design and methodology for a randomised controlled trial comparing annual and personalised variable-interval risk-based screening

Journal:	*BMJ Open*
Manuscript ID	bmjopen-2018-025788
Article Type:	Protocol
Date Submitted by the Author:	22-Aug-2018

Complete List of Authors:	Broadbent, Deborah; Royal Liverpool University Hospital, St Paul's Eye Unit;<br>Sampson, Christopher; Office of Health Economics, ;<br>Wang, Amu; University of Liverpool , Eye and Vision Science<br>Howard, Lola; University of Liverpool, Department of Biostatistics, ;<br>Williams, Abigail; University of Liverpool, Biostatistics<br>Howlin, Susan; Liverpool John Moores University School of Humanities and Social Science, Biostatistics<br>Appelbe, Duncan; University of Liverpool, Clinical Trials Research Centre<br>Moitt, Tracy; University of Liverpool Department of Biostatistics,<br>Cheyne, C; University of Liverpool, Biostatistics<br>Rahni, Mehrdad; Department of Eye and Vision Science, University of Liverpool, Institute of Ageing and Chronic Disease<br>Kelly, John; Royal Liverpool University Hospital, St Paul's Eye Unit<br>Collins, John; Royal Liverpool University Hospital, Department of Eye andVision Science<br>García-Fiñana, Marta; University of Liverpool, Biostatistics<br>Stratton, Irene; Gloucester Hospitals NHSFT, Gloucestershire Retinopathy research Group; home,<br>James, Marilyn; University of Nottingham, School of Medicine<br>Harding, Simon; Department of Eye and Vision Science, University of Liverpool, Institute of Ageing and Chronic Disease

Keywords:	Diabetic retinopathy < DIABETES & ENDOCRINOLOGY, General diabetes < DIABETES & ENDOCRINOLOGY, Health economics < HEALTH SERVICES ADMINISTRATION & MANAGEMENT, Health policy < HEALTH SERVICES ADMINISTRATION & MANAGEMENT, Medical ophthalmology < OPHTHALMOLOGY, Medical retina < OPHTHALMOLOGY

SCHOLARONE™
Manuscripts

Individualised Screening for Diabetic Retinopathy: the ISDR study. Rationale, design and methodology for a randomised controlled trial comparing annual and personalised variable-interval risk-based screening.

Authors

Deborah M Broadbent,[1,2*] Christopher J Sampson,[3] Amu Wang, [1] Lola Howard,[4] Abigail E Williams, [4], Susan U Howlin,[4] Duncan Appelbe,[4] Tracy Moitt,[4] Christopher P Cheyne,[4,5] Mehrdad Mobayen Rahni,[1] John Kelly,[6] John Collins,[6] Marta García-Fiñana,[4,5] Irene M Stratton,[7] Marilyn James,[3] Simon P Harding[1,2] for the ISDR Study Group.

Affiliations

1. Department of Eye and Vision Science, Institute of Ageing and Chronic Disease, University of Liverpool, UK
2. St. Pauls Eye Unit, Royal Liverpool University Hospital, UK
3. Division of Rehabilitation and Ageing, School of Medicine, University of Nottingham, UK
4. Department of Biostatistics, Clinical Trials Research Centre, University of Liverpool, UK
5. Department of Biostatistics, University of Liverpool, UK
6. ISDR Patient and Public Involvement Group
7. Gloucestershire Retinal Research Group, Cheltenham General Hospital, Cheltenham, UK

Correspondence:

Dr Deborah M Broadbent

Honorary Senior Lecturer,

Department of Eye and Vision Science,

For peer review only

3rd Floor University Clinical Departments

Royal Liverpool University Hospital

Prescot Street

Liverpool L7 8XP

Tel +44 151 706 2408

Fax +44 151 706 2409

deborah.broadbent@rlbuht.nhs.uk

Key words

Diabetic retinopathy, risk calculation engine, risk-based screening, cost-effectiveness, personalised medicine

Word count 4122

For peer review only

For peer review only - http://bmjopen.bmj.com/site/about/guidelines.xhtml

Abstract

Introduction

Currently all patients aged 12 years and over in the UK are invited for screening for diabetic retinopathy (DR) annually. Resources are not increasing despite a 5% increase in the numbers of patients with diabetes nationwide each year. We describe the rationale, design and methodology for a randomised controlled trial (RCT) evaluating the safety, acceptability and cost effectiveness of personalised variable-interval risk-based screening for DR. This is the first randomised trial of personalised screening for DR and the largest ophthalmic RCT in the UK.

Methods and analysis

Patients attending 7 screening clinics in the Liverpool Diabetic Eye Screening Programme (LDESP) were recruited into a single site RCT with a 1:1 allocation of patients to personalised variable-interval risk-based or annual screening intervals.

A Risk Calculation Engine developed for the trial estimates the probability that an individual will develop referable disease (screen positive DR) within the next 6, 12 or 24 months using demographic, retinopathy and systemic risk factor data from primary care and screening programme records. Dynamic, secure, real-time connections have been developed.

The primary outcome is attendance for follow-up screening. We will test for equivalence in attendance rates between the two arms. Secondary outcomes are rates and severity of DR, visual outcomes, cost effectiveness and health-related quality of life.

The required sample size was 4460 patients. Recruitment is complete and the trial is in follow-up.

Ethics and dissemination

For peer review only

For peer review only - http://bmjopen.bmj.com/site/about/guidelines.xhtml

Ethical approval was obtained from NRES Committee North West – Preston, reference 14/NW/0034. Results will be presented at international meetings and published in peer-reviewed journals. This pragmatic RCT will inform screening policy in the UK and elsewhere.

Strengths and limitations of the study

- RCT with cost-effectiveness
- Personalised medicine
- Substantial patient engagement
- Large recruitment target
- RCE requires populating with local data for generalisation to other locations

Trial registration number: ISRCTN 87561257

For peer review only

Introduction

Background

Diabetes mellitus (DM) is a life-long condition associated with the development of various macro- and microvascular complications, including diabetic retinopathy (DR) and maculopathy (a subgroup within DR). These are progressive conditions of the retina and macula, which can lead to visual impairment and blindness. After 20 years with diabetes, nearly all patients with type 1 [1] and over 60% with type 2 diabetes [2] will have some degree of DR.

DR affects people of all ages and is the commonest cause of blindness in people of working age in most developed countries worldwide. In the UK this position has changed. Liew et al reviewed the causes of blindness certifications in working age adults in England and Wales in 2009-2010 and reported that DR had dropped to the second commonest cause compared with ten years earlier [3]. Tighter control of glycaemia and hypertension will have contributed to this but it is likely that screening for DR has played a key role. There are an estimated 3.8 million people with diabetes aged 16 years and over in England [4], of whom almost 1 million are undiagnosed.

The early stages of DR (background and mild pre-proliferative) are asymptomatic and do not require any treatment. Sight threatening diabetic retinopathy (STDR) (an umbrella term that encompasses sight threatening levels of retinopathy and maculopathy) requires close monitoring by an ophthalmologist, and sometimes treatment, to prevent visual impairment (VI).

In the English National Diabetic Eye Screening Programme (NDESP) over 60 local programmes undertake primary care-based screening. In the LDESP fixed appointments are sent to patients as uptake rates are lower in open appointment programmes.

For peer review only

At screening appointments patients have their visual acuity (VA) measured, eye drops are administered to dilate pupils and then at least two 45° retinal digital images per eye are taken [5]. A National Grading Classification System has been implemented and patients with screen positive DR are referred to the Hospital Eye Service (HES) for assessment for STDR by an ophthalmologist.

Treatment (by laser photocoagulation or intravitreal injections) aims to stop progression and stabilise retinopathy. Treatment cannot always reverse the process once vision is lost. Screening is therefore recommended to identify STDR at the optimal time-point for treatment.

Systematic screening for DR was introduced across the UK by 2007 and annual screening for all people with diabetes aged twelve years or older was recommended. The one-year interval between screening invitations (screen interval) was based on expert opinion, rather than direct evidence. In 2016 the UK National Screening Committee (NSC) recommended that people with diabetes at low risk of sight loss could be screened every two years [6] but this has not yet been adopted. This stratified approach was based on evidence from a large observational study in one English programme, with validation in the Welsh, Scottish and NI programmes and 4 more English programmes and included a cost effectiveness analysis [7].

Available evidence show that many people at low risk of developing referable DR between annual screening appointments who could safely be screened less often [8], whilst others are at high risk and might benefit from more frequent screening [9]. Davies et al used simulation modelling on data from a literature survey of European and American populations in 1996 and concluded that biannual screening could be considered where patient compliance and screening sensitivities were both high [10]. Data on 10-year incidence from the Liverpool Diabetic Eye Study (LDES) in a population of patients with type

For peer review only - http://bmjopen.bmj.com/site/about/guidelines.xhtml

2 diabetes and enrolled in a systematic screening programme suggested that a 3-yearly screening interval could be adopted for patients with no retinopathy at baseline but yearly or more frequent screening was needed for patients with higher grades of retinopathy or insulin use [11]. Similar results were shown for type 1 DM [12]. In Sweden, biennial screen intervals have been used for some time for subjects without retinopathy [13]. A study carried out in Malmo prospectively followed patients with type 2 DM and no retinopathy and concluded that it appeared safe to adopt 3-year intervals [14] as suggested by the Liverpool group. However, this group of patients were compliant (only 9% did not attend for follow-up), had a short duration of diabetes (6 ± 6 years) and good control (HbA1c 6.4 ± 1.5% at baseline and 6.3 ± 1.3% at 3 year follow up).

Previous studies have shown that screening for DR is a highly cost-effective intervention [15, 16]. The higher the take-up rates for screening, the higher the cost effectiveness [17]. However resources are not increasing despite a 5% increase in the numbers of patients with diabetes nationwide each year.

Two studies have looked at stratified screening and shown that the risk of progression to STDR is significantly higher for those with background DR (BDR) in both eyes than those with BDR in only one or in neither eye [18, 19]. The first of these two studies suggested combining the results from 2 consecutive years of photographic screening enabled estimation of the risk of future development of STDR. Patients with no DR on two consecutive visits were deemed to be low risk.

So why hasn't an extended interval in screening for STDR been more widely adopted? There has been considerable concern about safety of extending screen intervals and no RCT to date [20]. The experience of Sweden and Scotland is reassuring but the populations are much smaller and generally better engaged. If people receive the impression that they are

For peer review only - http://bmjopen.bmj.com/site/about/guidelines.xhtml

at low risk they may disengage with other aspects of diabetes care. In spite of our own evidence from 2003, in the absence of the required safety data it appears unwise to recommend 2 or 3-year intervals for people with diabetes at low risk without further studies.

Rationale for a RCT

With this in mind we designed a RCT to investigate the safety of extending screening intervals in low risk patients. We included the emerging theories and technologies of risk prediction and personalisation to develop a variable-interval risk-based screening approach and added a health economics evaluation of cost-effectiveness.

In addition to severity of retinopathy, the risk of development and progression of DR to a level that requires treatment is related to age, gender [1,2] , duration of diabetes, HbA1c levels [21-26], blood pressure [27-29], lipid levels [30] and proteinuria [31]. If the contribution of each risk factor to overall risk (as well as the overall contribution in combination) could be calculated, and data on each risk factor were available for individuals, individual risks could be estimated and screening frequencies could be tailored to the level of personal risk [32].

We developed a risk calculation engine (RCE) using a longitudinal dataset from our local diabetic population to predict the risk of developing STDR, and have reported confident estimates of the risk of having STDR. [33]. In Iceland a similar 'risk engine' has also been developed to estimate the risk of development of STDR, based on modelling of historic epidemiological data on a limited number of risk factors for DR [34].

We embedded a patient and public involvement (PPI) group in the development of the RCE and subsequent design of the trial. There has been scant research into patients' understanding of screening and their views about introducing variable screening intervals.

For peer review only - http://bmjopen.bmj.com/site/about/guidelines.xhtml

Yeo et al (2012) reported that extended intervals may be acceptable to the majority of people with diabetes if there was adequate evidence to support such a change [35].

We found that over a series of workshops the patient volunteers became expert in the field and made important design decisions.

In this paper we report the key features of the design of our RCT. We believe that screening for referable DR at intervals based on individual risk could be both safe and cost-effective compared with annual screening.

Funding and ethics approval

The ISDR RCT is part of a larger programme of applied research funded by a £2.1m grant from the National Institute for Health Research (NIHR) Programme Grants for Applied Research (PGfAR) programme – reference RG-PG-1210-12016. SPH is the Chief Investigator for the 7 workstream programmes and DMB the Principal Investigator for the RCT. The RCT is supported by the Clinical Trials Research Centre (CTRC) in Liverpool. The study protocol [36], patient information sheets and consent forms have received ethical approval from the National Research Ethics Service Committee North West – Preston (reference: 14/NW/0034). Recruitment to the trial was supported by research staff from Clinical Research Network (CRN) North West Coast, LDESP staff and trained student volunteers.

Trial status

The trial opened to recruitment on the 12th November 2014. The first patient was randomised on the 19th November 2014. The trial was closed to enrolment on the 31 May 2016. The target of 4460 patients was reached. The Programme Grant completes on 30 April 2019.

For peer review only - http://bmjopen.bmj.com/site/about/guidelines.xhtml

Patient and Public Involvement

We have included members of our Patient and Public Information (PPI) Group in all stages of the design of the trial. Our PPI group consists of 7 individuals and has met at regular intervals throughout the lifetime of the trial. Individuals are active participants in the programme and trial steering committees, programme investigators committee and trial management committee. Individuals were involved in the concept and development of the research questions, design of the trial and the grant application. One patient is a co-investigator and was closely involved in designing the intervention including reviewing the potential burden. The group was involved in the choice of risk factors for consideration for the RCE and secondary outcome measures. They developed a set of patient centred outcomes, most notably adding visual impairment and need for treatment which they viewed as particularly important to patients. They considered the chance of missing disease if screen intervals were extended and after several structured sessions settled on a 2.5% risk as an acceptable limit. They were not involved in actual recruitment of patients but they visited the screening centres and observed recruitment and gave constructive comments which improved the process.

Regarding dissemination of results, these will be sent to the participants' General Practitioners. Participants have also been advised to let the researcher know if they would like a summary of the results themselves.

Participants, interventions and outcomes

Trial design

The ISDR RCT had 1:1 allocation to a personalised risk based screening recall (6, 12 or 24 months) or annual screening (current routine care).

For peer review only - http://bmjopen.bmj.com/site/about/guidelines.xhtml

Objectives

The objective of the ISDR RCT is to evaluate the safety, acceptability and cost-effectiveness of personalised risk-based screening in a whole population setting. The main aim is to assess the relative safety of variable-interval screening as measured by attendance rate. We will test the hypothesis that the difference in attendance rates between the two pathways is within an acceptable equivalence margin.

Study setting

This single site trial is being conducted in all 7 screening clinics in the Liverpool Diabetic Eye Screening Programme (LDESP), which is part of the English National Diabetic Eye Screening Programme (NDESP).

Study population

Patients with diabetes registered with a GP whose postcode is in the city boundaries of Liverpool and attending for screening for DR were invited to enter the trial. The inclusion and exclusion criteria are given in Table 1. The Schematic of the Study Design is given in Figure 1.

Data flows

Data flows are shown in Figure 2. Data on the patient's retinopathy status feeds automatically from the screening software (OptoMize) into the data warehouse and then to the randomisation and RCE programmes on a daily basis. The ISDR data warehouse automatically populates the majority of the fields in the baseline and follow-up electronic CRFs (OpenClinica), including data that allowed randomisation to occur.

Primary endpoint

The primary outcome is the attendance rate for follow-up screening in the two arms of the study. Non-attendance is defined as failure to attend any screening appointments within 90

For peer review only - http://bmjopen.bmj.com/site/about/guidelines.xhtml

For peer review only

days of the expected follow-up date, irrespective of how many appointments they had been sent.

Secondary endpoints

- Number of cases of STDR detected
- Retinopathy level at screening
- Maculopathy level at screening
- Number of false positive screening episodes
- Number of screening appointments
- Number of diabetes assessment clinic appointments
- Number of other eye appointments for DR
- Visual acuity (logMAR)
- New visual impairment (≥ +0.50 logMAR)
- New visual impairment (≥ +0.50 logMAR) which in the opinion of an experienced clinician is due to DR
- Number of missed appointments to screening
- Patient acceptability measures (using a questionnaire designed for the RCT)
- Quality-adjusted life years (QALYs) estimated using EQ-5D-5L and Health Utilities Index Mark 3 (HUI3)
- Cost per QALY

Intervention, assessments and procedures

The ISDR data warehouse (DW) is a relational database which stores both the data and how the data are related. The Liverpool GPs gave permission for their patients to be directly contacted to give permission for the data held in primary care relating to their diabetes to

For peer review only - http://bmjopen.bmj.com/site/about/guidelines.xhtml

be transferred to the DW. The DW was established to collate and dynamically link individual risk factor data from GP practices, STDR outcome data from the DR assessment clinic at St Pauls Eye Unit and the LDESP screening software (OptoMize). This central data repository was used to develop the locally applicable risk calculation engine (RCE) described above using a continuous Markov model.

A risk calculation engine (RCE) has been developed using routinely collected primary and secondary care data from 11,806 people with diabetes (46,525 screening episodes) in the LDESP. A detailed description of the RCE has already been published [33]. In brief, the final covariates for the RCE were selected using a 3-step decision process. A number of risk factors for developing STDR were identified in the published literature and in preliminary work in Liverpool. These potential covariates were reviewed by the clinical team and the ISDRPPI group and potential additional covariates were suggested. Potential covariates with a missingness rate of less than 20% in the data warehouse were identified and removed. Then a statistical evaluation of the predictive value of each remaining covariate was undertaken. The covariates selected were disease state (current retinopathy levels in both eyes); age; duration of diabetes; glycated haemoglobin; systolic blood pressure; and total cholesterol.

Our PPI group defined the degree of risk (up to 2.5% risk of developing screen positive diabetic retinopathy) acceptable to people with diabetes, allowing assignment of individuals to screen intervals at the time points 6, 12 or 24 months. The choice of these intervals was based on a review of the available literature, our data and consensus between the PPI and research teams. Following each negative screening outcome, patients are assigned to the longest recall period up to 24 months at which their risk estimation would not exceed the 2.5% threshold.

For peer review only - http://bmjopen.bmj.com/site/about/guidelines.xhtml

Consent

Patients received a Patient Information Leaflet with all the details of the trial with their screening appointment reminder letter. On attending the screening clinic patients were approached by trained researchers to establish whether they wished to participate in the trial. If the patient expressed and interest the researcher took written informed consent and enrolled them into the trial. If a patient subsequently requested to be withdrawn from the trial they reverted to routine care. Reasons for, and level of, withdrawal were collected.

Randomisation

Randomisation could only be completed once the patient's retinal images had been graded, as it was only possible at this point to complete the inclusion and exclusion criteria. Patients who screened positive due to DR, other eye disease or ungradeable images were therefore consented but were not included in the trial.

Eligible patients were randomly allocated to the personalised risk-based screening recall or the usual fixed annual interval. Block randomisation with stratification was used to ensure adequate representation in each arm of the trial for groups where there are small numbers of participants (such as children aged 12-15). The screening clinics in Liverpool had different proportions of patients in hard to reach groups, such as minority ethnic groups, attendance rates and social deprivation indices.

After randomisation the RCE was used to generate the individual's recall period for patients in the personalised arm of the trial and the data was sent back to the screening software.

Study visits and assessments

At each follow-up visit, patients randomised to the personalised risk-based screening arm have their risk re-calculated and the next recall interval determined accordingly.

Health economics

For peer review only - http://bmjopen.bmj.com/site/about/guidelines.xhtml

A health economics questionnaire, incorporating the health-related quality of life (QoL) questionnaires EQ-5D-5L [37] and Health Utilities Index Mark 3 (HUI3) [38] and a bespoke visit questionnaire [39], were completed by the first 868 eligible patients enrolled into the study (a minimum of 700 patients was required to provide sufficient data). The QoL questionnaires are repeated at every subsequent follow-up visit for those patients who completed it at baseline.

For peer review only

For peer review only - http://bmjopen.bmj.com/site/about/guidelines.xhtml

Trial Intervention diagram

Procedures	*Screening*	*Baseline*	*Randomisation*	*Follow-Up Schedule* *6 months***	*12 months***	*18 months***	*24 months***	*30 months***	*36 months***	*Study Completion*	*Premature Discontinuation*
Signed Consent Form	*X*										
Visual Acuity(LogMar)		*X*		*X*	*X*	*X*	*X*	*X*	*X*		
Visit Questionnaire		*X*									
*QoL Questionaire** *(HUI3 and EQ5D)*		*X*		*X*	*X*	*X*	*X*	*X*	*X*		
Assessment of Eligibility Criteria	*X*	*X*	*X*								
Randomisation			*X*								
Digital Imaging		*X*		*X*	*X*	*X*	*X*	*X*	*X*		
Acceptability Questionnaire									*X*		

*The EQ-5D-5L, HUI3 data will be collected on around 100-200 eligible patients and repeated at each visit for those individual patients. Data will be stratified to balance equal numbers of patients in each arm and representation from all screening sites.

**Patients are randomised to 2 arms. In one arm patients attend at 12 month intervals. In the other arm patients attend at 6, 12 or 24 monthly intervals.

Sample size and planned analyses

The primary aim is to test for equivalence in attendance rates between personalised risk-based screening and annual screening. The analysis will test the hypothesis that the difference in attendance rates between the two pathways is within an acceptable range (5% equivalence margin).

The estimated minimum number of patients required with 90% power, 5% significance level and an equivalence margin of 5%, is 3940. Taking into account a loss to follow-up rate of 6% due to death and other exclusion from screening (note that non-attendance is the primary

For peer review only

For peer review only - http://bmjopen.bmj.com/site/about/guidelines.xhtml

outcome and therefore is not factored here) the target for recruitment (randomised into the trial) was 4460 patients.

A secondary aim is to investigate whether personalised screening can be considered as non-inferior in detection of STDR when compared to annual screening. The STDR detection rate predicted for the usual care pathway during the two years follow-up is approximately 6%, based on data from the Liverpool DESP. The sample size required to address the first question (n=4460 patients randomised with 3940 patients retained after 2 years from baseline) will permit us to test for non-inferiority in STDR detection with a maximum allowable reduction of 1.5% of the personalised care pathway compared to standard care, with 5% significance level and power between 60-65%.

We will undertake sub-group analyses to assess differences in attendance rates between the two arms for the three different retinopathy groups (patients with no retinopathy in either eye, patients with retinopathy in one eye and patients with retinopathy in both eyes when the overall risk of developing STDR is calculated). A mixed-effects model that takes into account the patient's covariate information over time (level of cholesterol, HbA1c, etc.), a logistic model for STDR detection and a survival model with time-dependent random effects that accounts for the clinical and demographic characteristics of non-attenders will be jointly fitted. A Gaussian latent variable that encapsulates the underlying state of health of the patient not explained by the observable covariates will connect the three models.

We will evaluate the cost-effectiveness of personalised risk-based screening compared with annual screening. Using screening activity data stored in the ISDR DW and information collected with the visit questionnaire and EQ-5D-5L and HUI3 responses, we will estimate the cost-per-QALY associated with risk-based and annual screening from a NHS perspective.

For peer review only - http://bmjopen.bmj.com/site/about/guidelines.xhtml

We will present incremental cost-effectiveness ratios and cost-effectiveness acceptability curves to characterise the uncertainty associated with our estimates.

We will apply sensitivity analyses to check the sensitivity of the results on the assumption that missing data are missing at random. Different scenarios for missing-data mechanisms will be explored.

Conclusions

This paper describes the design of a RCT to evaluate the feasibility, safety, acceptability and cost-effectiveness of individualised variable-intervals risk-based screening compared to fixed annual interval screening. Safety will be measured by the effect on attendance rates to screening, rates and severity of DR, visual outcomes and impact on general diabetes care. As far as we are aware this is the only study to investigate the impact of implementing personalised screening for diabetic retinopathy in an RCT.

Authors' contributions

DMB (Trial PI) and SPH (CI) conceived the trial questions and have been involved in all stages of the study design, and together with AEW (Trial Coordinator) and AW (ISDR Programme Manager) participated in writing the protocol, submission to the funding body and application to the ethics committee. SUH is a Senior Data Manager for the CTRC and DA is the information Systems Manager for the CTRC. Both contributed to writing the protocol and devised the electronic CRF system and data monitoring systems. MG-F is the lead statistician and co-investigator and CPC is the trial statistician. IMS is a leading UK statistician. All participated in writing the protocol and statistical aspects of the study. MMR is the data warehouse manager. MJ is the lead health economist and a co-investigator and CJS is the trial health economist. They designed the heath economic analysis. TM is a senior

For peer review only - http://bmjopen.bmj.com/site/about/guidelines.xhtml

trials manager and a co-investigator. LH replaced AEW in the CTRC and has been involved in revising the protocol and preparing the manuscript with DMB and CJS. JK and JC are PPI members and contributed to the design of the RCE and the trial. All authors read and approved the final manuscript.

Legends

Figure 1: Schematic of trial design

Figure 2: Data Flows In step 1 data for consented participents is requested from EMIS, passed to the Data Warehouse and cleaned prior to storage. In step 2 data is exchanged between the data warehouse and Optomize (subjects whose risk needs to be calculated are sent to the data warehouse, subjects for whom their risk and therefore recall interval has been calculated is returned to optomise for appointment letter generation). In step 3 the participants risk is calculated (when all the covariates are available), if randomisation is required they are then randomised. The data is then stored in the study database and that required to be returned to the data warehouse. All processes in steps 1 and 2 involve identifiable data, the processes in step 3 all use pseudanonomised data for security reasons (the trial team with access to the trial database do not have a need to see raw identifiers). Step 1 occurs on a bi-monthy period, steps 2 & 3 occur on a daily basis, under ideal conditions it takes three days for the data to pass through all parts of steps 1 and 2 – this is due to an airgap (manual transfer) at step 2 between the data warehouse and the NHS systems.

Table 1: Inclusion and exclusion criteria in the individualised variableinterval risk-based screening for diabetic retinopathy randomised controlled trial.

Competing interests

For peer review only - http://bmjopen.bmj.com/site/about/guidelines.xhtml

The authors report no competing interests

Funding statement

This paper presents independent research funded by the National Institute for Health Research (NIHR) under the Programme Grants for Applied Research programme (RP-PG-1210-12016). The views expressed are those of the authors and not necessarily those of the NHS, the NIHR or the Department of Health.

Data sharing statement

The formal protocol is published at 2014 DOI: 10.6084/m9.figshare.5053336.v1

Trial sponsor

University of Liverpool Research Support Office,

1st floor Duncan Building,

Daulby Street,

Liverpool L69 3GA.

Tel: 0151 706 3702

ISDR Study Group

Simon P Harding (Study Group Chair), Deborah M Broadbent (Trial PI), Anthony C Fisher, Mark Gabbay, Marta García-Fiñana, Marilyn James, Tracy Moitt, John R Roberts, Daniel Seddon, Irene M Stratton, Paula Williamson, Duncan Appelbe, Lola Howard, Susan U Howlin, Ayesh Alshukri, Abigail E Williams, Christopher P Cheyne, Antonio Eleuteri, Christopher Grierson, Mehrdad Mobayen-Rahni, Christopher J Sampson, David Szmyt, Clare Thetford, Maria Pilar Vazquez Arango, Amu Wang, Helen Cooper, John Collins, John Kelly, Nathalie Massat, Gideon Smith, Vineeth Kumar, Chris Rogers, Julia West, Naveed Younis, Peter Lees, Sandra Lees, Emily Doncaster, Betty Williams, Ticiana Criddle, Stephanie Perrett.

For peer review only - http://bmjopen.bmj.com/site/about/guidelines.xhtml

For peer review only

Acknowledgments

We would like to acknowledge the outstanding attention to detail from the ISDR administrative team and the CTRC. We would like to thank the North West Coast CRN and our other trained researchers for their invaluable help in recruiting to target. We would also like to thank the LDESP for their support and assistance. We would particularly like to thank our PPI members for their invaluable advice and enthusiastic support for the study.

Christopher J Sampson is currently affiliated to the Office of Health Economics, Southside, 7th Floor, 105 Victoria Street, London SW1E 6QT

References

1. Klein R, Klein BE, Moss SE, Davis MD, DeMets DL. The Wisconsin epidemiologic study of diabetic retinopathy. II. Prevalence and risk of diabetic retinopathy when age at diagnosis is less than 30 years. *Arch Ophthalmol* 1984, 102:520-526.

2. Klein R, Klein BE, Moss SE, Davis MD, DeMets DL. The Wisconsin epidemiologic study of diabetic retinopathy. III. Prevalence and risk of diabetic retinopathy when age at diagnosis is 30 or more years. *Arch Ophthalmol* 1984, 102:527-532

3. Liew, G, Michaelides, M, Bunce, C. A comparison of the causes of blindness certifications in England and Wales in working age adults (16 to 64 years), 1999 to 2000 with 2009 to 2010. BMJ Open 2014, 12 February 2014

4. PHE bulletin 2016 https://www.gov.uk/government/uploads/system/uploads/attachment_data/file/556910/PHE_Bulletin_September_2016.pdf Last accessed 03.01.18

For peer review only - http://bmjopen.bmj.com/site/about/guidelines.xhtml

5. NHS diabetic eye screening programme. Diabetic eye screening: commission and provide. https://www.gov.uk/government/collections/diabetic-eye-screening-commission-and-provide Last accessed 03.01.18

6. UK National Screening Committee. The UK NSC recommendation on Diabetic Retinopathy screening in adults. January 2016. Available at http://legacy.screening.nhs.uk/diabeticretinopathy. Last accessed 03.01.18

7. Scanlon PH, Aldington, SJ, Leal J, Luengo-Fernandez R, Oke J, Sivaprasad S et al. Development of a cost-effectiveness model for optimisation of the screening interval in diabetic retinopathy screening. Health Technol Assess 2015;19:1-116

8. Taylor-Phillips S, Mistry H, Leslie R, Todkill D, Tsertd vadze A, Connock M et al. Extending the diabetic retinopathy screening interval beyond 1 year: systematic review. Br J Ophthalmol 2016;100:105-114

9. Leese GP, Stratton IM, Land M, Bachmann MO, Jones C, Scanlon P et al. Progressio of diabetes retinal status within community screening programs and potential implications for screening intervals. Diabetes Care 2015;38:488-494

10. Davies R, Sullivan P, Canning C. Simulation of diabetic eye disease to compare screening policies. *British Journal of Ophthalmology* 1996, 80:945-950.

11. Younis N, Broadbent DM, Vora JP, Harding SP. Incidence of sight-threatening retinopathy in patients with Type 2 diabetes in the Liverpool Diabetic Eye Study: a cohort study. *Lancet* 2003, 361:195-200

12. Younis N, Broadbent DM, Harding SP, Vora JP. Incidence of sight-threatening retinopathy in Type 1 diabetes in a systematic screening programme. *Diabet Med* 2003, 20:758-765

For peer review only - http://bmjopen.bmj.com/site/about/guidelines.xhtml

13. Stefansson E, Bek T, Porta M, Larsen N, Kristinsson JK, Agardh E. Screening and prevention of diabetic blindness. *Acta Ophthalmol Scand* 2000;78:374-385

14. Agardh E, Tabayat-Khani P. Adopting 3-year screening intervals for sight-threatening retinal vascular lesions in Type 2 diabetic subjects without retinopathy. *Diab Care* 2011;34:1-2

15. Javitt JC, Aiello LP: Cost-Effectiveness of Detecting and Treating Diabetic Retinopathy. *Annals of Internal Medicine* 1996, 124:164-169.

16. Jones S, Edwards RT. Diabetic retinopathy screening: a systematic review of the economic evidence. *Diabet Med* 2010, 27(3):249–56. DOI: 10.1111/j.1464-5491.2009.02870.x

17. James M, Turner DA, Broadbent DM, Vora J, Harding SP. Cost effectiveness analysis of screening for sight threatening diabetic eye disease. *BMJ* 2000, 320(7250):1627–31

18. Stratton IM, Aldington SJ, Taylor DJ, Adler AI, Scanlon PH. A simple risk stratification for time to development of sight-threatening diabetic retinopathy. Diabetes Care. 2013 Mar; 36(3):580-5. DOI: 10.2337/dc12-0625

19. Scanlon PH, Stratton IM, Histed M, Chave SJ, Aldington SJ. The influence of background diabetic retinopathy in the second eye on rates of progression of diabetic retinopathy between 2005 and 2010. Acta Ophthalmol. 2013 Aug; 91(5):e335-9. DOI: 10.1111/aos.12074

20. Taylor-Phillips S, Mistry H, Leslie R et al. Extending the diabetic retinopathy screening interval beyond 1 year: systematic review. http://dx.doi.org/10.1136/bjophthalmol-2014-305938

For peer review only - http://bmjopen.bmj.com/site/about/guidelines.xhtml

21. The Diabetes Control and Complications Trial Research Group. The effect of intensive treatment of diabetes on the development and progression of long-term complications in insulin-dependent diabetes mellitus. *N Engl J Med* 1993, 329:977-986

22. Davis MD, Fisher MR, Gangnon RE, Barton F, Aiello LM, Chew EY, Ferris FL, 3rd, Knatterud GL. Risk factors for high-risk proliferative diabetic retinopathy and severe visual loss: Early Treatment Diabetic Retinopathy Study Report #18. *Invest Ophthalmol Vis Sci* 1998, 39:233-252

23. Marshall G, Garg SK, Jackson WE, Holmes DL, Chase HP. Factors influencing the onset and progression of diabetic retinopathy in subjects with insulin-dependent diabetes mellitus. *Ophthalmology* 1993, 100:1133-1139

24. Stratton IM, Kohner EM, Aldington SJ, Turner RC, Holman RR, Manley SE, Matthews DR: UKPDS 50. Risk factors for incidence and progression of retinopathy in Type II diabetes over 6 years from diagnosis. *Diabetologia* 2001, 44:156-163

25. Agardh CD, Agardh E, Torffvit O. The association between retinopathy, nephropathy, cardiovascular disease and long-term metabolic control in Type 1 diabetes mellitus: a 5 year follow-up study of 442 adult patients in routine care. *Diabetes Res Clin Pract* 1997, 35:113-121

26. Vijan S, Hofer TP, Hayward RA. Estimated Benefits of Glycemic Control in Microvascular Complications in Type 2 Diabetes. *Annals of Internal Medicine* 1997, 127:788-795

27. UK Prospective Diabetes Study Group. Tight blood pressure control and risk of macrovascular and microvascular complications in Type 2 diabetes: UKPDS 38. . *BMJ* 1998, 317:703-713

For peer review only - http://bmjopen.bmj.com/site/about/guidelines.xhtml

28. Gallego PH, Craig ME, Hing S, Donaghue KC. Role of blood pressure in development of early retinopathy in adolescents with Type 1 diabetes: prospective cohort study. *BMJ* 2008, 337:a918

29. Chaturvedi N, Sjolie AK, Stephenson JM, Abrahamian H, Keipes M, Castellarin A, Rogulja-Pepeonik Z, Fuller JH. Effect of lisinopril on progression of retinopathy in normotensive people with Type 1 diabetes. The EUCLID Study Group. EURODIAB Controlled Trial of Lisinopril in Insulin-Dependent Diabetes Mellitus. *Lancet* 1998, 351:28-31

30. Keech A, Simes RJ, Barter P, Best J, Scott R, Taskinen MR, Forder P, Pillai A, Davis T, Glasziou P, et al. Effects of long-term fenofibrate therapy on cardiovascular events in 9795 people with Type 2 diabetes mellitus (the FIELD study): randomised controlled trial. *Lancet* 2005, 366:1849-1861

31. Klein R, Klein BEK, Moss SE, Cruickshanks KJ. The Wisconsin epidemiologic study of diabetic retinopathy: XVII: The 14-year incidence and progression of diabetic retinopathy and associated risk factors in Type 1 diabetes. *Ophthalmology* 1998, 105:1801-1815

32. Sampson CJ, James M, Broadbent DM, Harding SP. Stratifying the NHS Diabetic Eye Screening Programme: into the unknown? Diabet Med 2016, 33(12): 1612–4. DOI: 10.1111/dme.13192

33. Eleuteri A, Fisher AC, Broadbent DM, García-Fiñana M, Cheyne CP, Wang A, Stratton IM, Gabbay M, Seddon D, Harding SP for the ISDR Study Group. Individualised variable interval risk-based screening for sight threatening diabetic retinopathy – the Liverpool Risk Calculation Engine. *Diabetologia* 2017; 60(11): 2174-2182

34. Aspelund T, Porisdottir O, Olafsdottir E, Gudmundsdottir A, Einarsdottir AB, Mehlsen J, Einarsson S, Palsson O, Bek T, Stefansson E. Individual risk assessment and information

For peer review only

For peer review only - http://bmjopen.bmj.com/site/about/guidelines.xhtml

technology to optimise screening frequency for diabetic retinopathy. *Diabetologia* 2011, DOI: 10.1007/s00125-011-2257-7

35. Yeo ST, Edwards RT, Luzio SD, Charles JM, Thomas RL, Peters JM, et al. Diabetic retinopathy screening: perspectives of people with diabetes, screening intervals and costs of attending screening. Diabet Med 2012, 29(7):878–85. DOI: 10.1111/j.1464-5491.2012.03637.x

36. Broadbent DM, Bennett A, Moitt T, Howlin S, James M, Sampson C, et al. Introducing personalised risk based intervals in screening for diabetic retinopathy: development, implementation and assessment of safety, cost-effectiveness and patient experience. Workstream E: Randomised trial comparing standard and test screening intervals protocols. 2014 DOI: 10.6084/m9.figshare.5053336.v1

37. Herdman M, Gudex C, Lloyd A, Janssen M, Kind P, Parkin D, et al. Development and preliminary testing of the new five-level version of EQ-5D (EQ-5D-5L). Qual Life Res 2011, 20(10):1727–36. DOI: 10.1007/s11136-011-9903-x

38. Feeny D, Furlong W, Boyle M, Torrance GW. Multi-attribute health status classification systems. Health Utilities Index. Pharmacoeconomics 1995, 7(6):490–502. DOI: 10.2165/00019053-199507060-00004

39. James M, Sampson C. ISDR Visit Questionnaire. Database of Instruments for Resource Use Measurement. 2015. DOI: Available from: http://dirum.org/instruments/details/90 DOI: 10.6084/m9.figshare.1301022.v1

For peer review only

For peer review only

For peer review only - http://bmjopen.bmj.com/site/about/guidelines.xhtml

Table 1: Inclusion and exclusion criteria in the Individualised variableinterval risk-based Screening for Diabetic Retinopathy randomised controlled trial.

**Inclusion Criteria**	**Exclusion Criteria**
adults, young people and children who are aged ≥12 years	under age 12 years
due to be offered an appointment for retinal screening during the recruitment period	
registered with a participating GP practice	are not registered with a participating GP practice
are included in the study data warehouse (have not opted out)	have opted out from the study data warehouse
have no retinopathy or have retinopathy and maculopathy less than the definition of screen positive diabetic retinopathy	have screen positive diabetic eye disease or significant other eye disease requiring referral to the HES
have gradeable digital retinal images in both eyes	are ineligible for screening for whatever reason, including having ungradable digital retinal images, which includes patients who have only one eye or an ungradable eye with no visual potential
give their informed consent for participation	do not give consent for participation in the RCT
are not involved in any trial investigating a treatment aiming at preventing or modifying the development of STDR	are involved in any trial investigating a treatment aiming at preventing or modifying the development of STDR

For peer review only

Figure 1

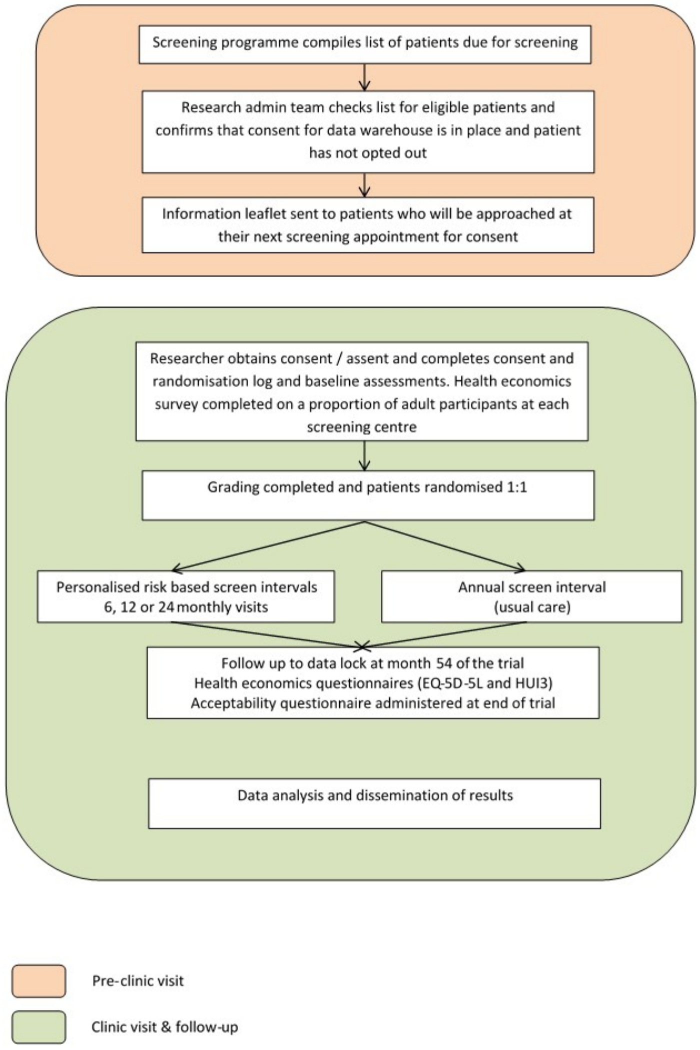

196x285mm (96 x 96 DPI)

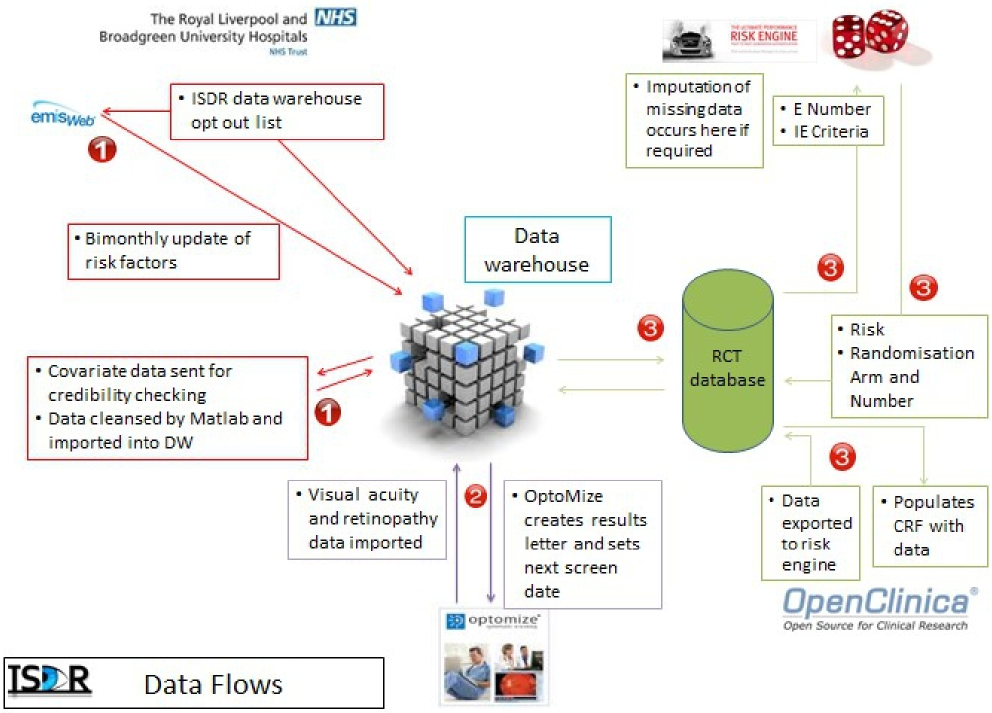

177x133mm (96 x 96 DPI)

BMJ Open

Individualised Screening for Diabetic Retinopathy: the ISDR study. Rationale, design and methodology for a randomised controlled trial comparing annual and personalised variable-interval risk-based screening

Journal:	*BMJ Open*
Manuscript ID	bmjopen-2018-025788.R1
Article Type:	Protocol
Date Submitted by the Author:	16-Nov-2018

Complete List of Authors:	Broadbent, Deborah; Royal Liverpool University Hospital, St Paul's Eye Unit;<br>Sampson, Christopher; Office of Health Economics, ;<br>Wang, Amu; University of Liverpool , Eye and Vision Science<br>Howard, Lola; University of Liverpool, Department of Biostatistics, ;<br>Williams, Abigail; University of Liverpool, Biostatistics<br>Howlin, Susan; Liverpool John Moores University School of Humanities and Social Science, Biostatistics<br>Appelbe, Duncan; University of Liverpool, Clinical Trials Research Centre<br>Moitt, Tracy; University of Liverpool Department of Biostatistics,<br>Cheyne, C; University of Liverpool, Biostatistics<br>Rahni, Mehrdad; Department of Eye and Vision Science, University of Liverpool, Institute of Ageing and Chronic Disease<br>Kelly, John; Royal Liverpool University Hospital, St Paul's Eye Unit<br>Collins, John; Royal Liverpool University Hospital, Department of Eye andVision Science<br>García-Fiñana, Marta; University of Liverpool, Biostatistics<br>Stratton, Irene; Gloucester Hospitals NHSFT, Gloucestershire Retinopathy research Group; home,<br>James, Marilyn; University of Nottingham, School of Medicine<br>Harding, Simon; Department of Eye and Vision Science, University of Liverpool, Institute of Ageing and Chronic Disease

**Primary Subject Heading**:	Ophthalmology
Secondary Subject Heading:	Diabetes and endocrinology, Health economics, Health services research, Public health, Patient-centred medicine
Keywords:	Diabetic retinopathy < DIABETES & ENDOCRINOLOGY, General diabetes < DIABETES & ENDOCRINOLOGY, Health economics < HEALTH SERVICES ADMINISTRATION & MANAGEMENT, Health policy < HEALTH SERVICES ADMINISTRATION & MANAGEMENT, Medical ophthalmology < OPHTHALMOLOGY, Medical retina < OPHTHALMOLOGY

SCHOLARONE™
Manuscripts

Individualised Screening for Diabetic Retinopathy: the ISDR study. Rationale, design and methodology for a randomised controlled trial comparing annual and personalised variable-interval risk-based screening.

Authors

Deborah M Broadbent,[1,2*] Christopher J Sampson,[3] Amu Wang,[1] Lola Howard,[4] Abigail E Williams,[4], Susan U Howlin,[4] Duncan Appelbe,[4] Tracy Moitt,[4] Christopher P Cheyne,[4,5] Mehrdad Mobayen Rahni,[1] John Kelly,[6] John Collins,[6] Marta García-Fiñana,[4,5] Irene M Stratton,[7] Marilyn James,[3] Simon P Harding[1,2] for the ISDR Study Group.

Affiliations

1. Department of Eye and Vision Science, Institute of Ageing and Chronic Disease, University of Liverpool, UK
2. St. Pauls Eye Unit, Royal Liverpool University Hospital, UK
3. Division of Rehabilitation and Ageing, School of Medicine, University of Nottingham, UK
4. Department of Biostatistics, Clinical Trials Research Centre, University of Liverpool, UK
5. Department of Biostatistics, University of Liverpool, UK
6. ISDR Patient and Public Involvement Group
7. Gloucestershire Retinal Research Group, Cheltenham General Hospital, Cheltenham, UK

Correspondence:

Dr Deborah M Broadbent

Honorary Senior Lecturer,

Department of Eye and Vision Science,

For peer review only

3rd Floor University Clinical Departments

Royal Liverpool University Hospital

Prescot Street

Liverpool L7 8XP

Tel +44 151 706 2408

Fax +44 151 706 2409

deborah.broadbent@rlbuht.nhs.uk

Key words

Diabetic retinopathy, risk calculation engine, risk-based screening, cost-effectiveness, personalised medicine

Word count 3854

For peer review only

Abstract

Introduction

Currently all people with diabetes (PWD) aged 12 years and over in the UK are invited for screening for diabetic retinopathy (DR) annually. Resources are not increasing despite a 5% increase in the numbers of PWD nationwide each year. We describe the rationale, design and methodology for a randomised controlled trial (RCT) evaluating the safety, acceptability and cost effectiveness of personalised variable-interval risk-based screening for DR. This is the first randomised trial of personalised screening for DR and the largest ophthalmic RCT in the UK.

Methods and analysis

PWD attending 7 screening clinics in the Liverpool Diabetic Eye Screening Programme (LDESP) were recruited into a single site RCT with a 1:1 allocation to personalised variable-interval risk-based or annual screening intervals.

A Risk Calculation Engine developed for the trial estimates the probability that an individual will develop referable disease (screen positive DR) within the next 6, 12 or 24 months using demographic, retinopathy and systemic risk factor data from primary care and screening programme records. Dynamic, secure, real-time connections have been developed.

The primary outcome is attendance for follow-up screening. We will test for equivalence in attendance rates between the two arms. Secondary outcomes are rates and severity of DR, visual outcomes, cost effectiveness and health-related quality of life.

The required sample size was 4460 PWD. Recruitment is complete and the trial is in follow-up.

Ethics and dissemination

For peer review only

Ethical approval was obtained from NRES Committee North West – Preston, reference 14/NW/0034. Results will be presented at international meetings and published in peer-reviewed journals. This pragmatic RCT will inform screening policy in the UK and elsewhere.

Strengths and limitations of the study

- RCT with cost-effectiveness
- Personalised medicine
- Substantial t engagement of PWD
- Large recruitment target
- RCE requires populating with local data for generalisation to other locations

Trial registration number: ISRCTN 87561257

For peer review only

Introduction

Background

Diabetes mellitus (DM) is a life-long condition associated with the development of various macro- and microvascular complications, including diabetic retinopathy (DR) and maculopathy (a subgroup within DR). These are progressive conditions of the retina and macula, which can lead to visual impairment and blindness. After 20 years with diabetes, nearly all people with type 1 diabetes [1] and over 60% with type 2 diabetes [2] will have some degree of DR.

DR affects people of all ages and is the commonest cause of blindness in people of working age in most developed countries worldwide. In the UK this position has changed. Liew et al reviewed the causes of blindness certifications in working age adults in England and Wales in 2009-2010 and reported that DR had dropped to the second commonest cause compared with ten years earlier [3]. Tighter control of glycaemia and hypertension will have contributed to this but it is likely that screening for DR has played a key role. There are an estimated 3.8 million people with diabetes (PWD) aged 16 years and over in England [4], of whom almost 1 million are undiagnosed.

The early stages of DR (background and mild pre-proliferative) are asymptomatic and do not require any treatment. Sight threatening diabetic retinopathy (STDR) (an umbrella term that encompasses sight threatening levels of retinopathy and maculopathy) requires close monitoring by an ophthalmologist, and sometimes treatment, to prevent visual impairment (VI).

In the English National Diabetic Eye Screening Programme (NDESP) over 60 local programmes undertake primary care-based screening. In the LDESP fixed appointments are sent to PWD as uptake rates are lower in open appointment programmes.

For peer review only

At screening appointments PWD have their visual acuity (VA) measured, eye drops are administered to dilate pupils and then at least two 45° retinal digital images per eye are taken [5]. A National Grading Classification System has been implemented and patients with screen positive DR are referred to the Hospital Eye Service (HES) for assessment for STDR by an ophthalmologist.

Treatment (by laser photocoagulation or intravitreal injections) aims to stop progression and stabilise retinopathy. Treatment cannot always reverse the process once vision is lost. Screening is therefore recommended to identify STDR at the optimal time-point for treatment.

Systematic screening for DR was introduced across the UK by 2007 and annual screening for all PWD aged twelve years or older was recommended. The one-year interval between screening invitations (screen interval) was based on expert opinion, rather than direct evidence. In 2016 the UK National Screening Committee (NSC) recommended that PWD at low risk of sight loss could be screened every two years [6] but this has not yet been adopted. This stratified approach was based on evidence from a large observational study in one English programme, with validation in the Welsh, Scottish and NI programmes and 4 more English programmes and included a cost effectiveness analysis [7]. Stratified screening is now due to be implemented in Scotland.

Available evidence shows that many people -at low risk of developing referable DR between annual screening appointments could safely be screened less often [8], whilst others are at high risk and might benefit from more frequent screening [9]. Davies et al used simulation modelling on data from a literature survey of European and American populations in 1996 and concluded that biannual screening could be considered where patient compliance and screening sensitivities were both high [10]. Data on 10-year incidence from the Liverpool

For peer review only

Diabetic Eye Study (LDES) in a population of people with type 2 diabetes and enrolled in a systematic screening programme suggested that a 3-yearly screening interval could be adopted for PWD with no retinopathy at baseline but yearly or more frequent screening was needed for people with higher grades of retinopathy or insulin use [11]. Similar results were shown for type 1 DM [12]. In Sweden, biennial screen intervals have been used for some time for subjects without retinopathy [13]. A study carried out in Malmo prospectively followed people with type 2 DM and no retinopathy and concluded that it appeared safe to adopt 3-year intervals [14] as suggested by the Liverpool group. However, this group of PWD were compliant (only 9% did not attend for follow-up), had a short duration of diabetes (6 ± 6 years) and good control (HbA1c 6.4 ± 1.5% at baseline and 6.3 ± 1.3% at 3 year follow up).

Previous studies have shown that screening for DR is a highly cost-effective intervention [15, 16]. The higher the take-up rates for screening, the higher the cost effectiveness [17]. However resources are not increasing despite a 5% increase in the numbers of PWD nationwide each year.

Two studies have looked at stratified screening and shown that the risk of progression to STDR is significantly higher for those with background DR (BDR) in both eyes than those with BDR in only one or in neither eye [18, 19]. The first of these two studies suggested combining the results from 2 consecutive years of photographic screening enabled estimation of the risk of future development of STDR. People with no DR on two consecutive visits were deemed to be low risk.

So why hasn't an extended interval in screening for STDR been more widely adopted? There has been considerable concern about safety of extending screen intervals and no RCT to date [20]. The experience of Sweden and Scotland is reassuring but the populations are

For peer review only

much smaller and generally better engaged. If people receive the impression that they are at low risk they may disengage with other aspects of diabetes care. In spite of our own evidence from 2003, in the absence of the required safety data it appears unwise to recommend 2 or 3-year intervals for people with diabetes at low risk without further studies.

Rationale for a RCT

With this in mind we designed a RCT to investigate the safety of extending screening intervals in low risk PWD. We included the emerging theories and technologies of risk prediction and personalisation to develop a variable-interval risk-based screening approach and added a health economics evaluation of cost-effectiveness.

In addition to severity of retinopathy, the risk of development and progression of DR to a level that requires treatment is related to age, gender [1,2] , duration of diabetes, HbA1c levels [21-26], blood pressure [27-29], lipid levels [30] and proteinuria [31]. If the contribution of each risk factor to overall risk (as well as the overall contribution in combination) could be calculated, and data on each risk factor were available for individuals, individual risks could be estimated and screening frequencies could be tailored to the level of personal risk [32].

We developed a risk calculation engine (RCE) using a longitudinal dataset from our local diabetic population to predict the risk of developing STDR, and have reported confident estimates of the risk of having STDR. [33]. In Iceland a similar ‘risk engine’ has also been developed to estimate the risk of development of STDR, based on modelling of historic epidemiological data on a limited number of risk factors for DR [34]. In the Netherlands a screening model based on patients’ risk has been validated in 3319 people with type 2 diabetes as part of the Hoorn Study. [35]

For peer review only

We embedded a patient and public involvement (PPI) group in the development of the RCE and subsequent design of the trial. There has been scant research into people's understanding of screening and their views about introducing variable screening intervals. Yeo et al (2012) reported that extended intervals may be acceptable to the majority of PWD if there was adequate evidence to support such a change [36].

We found that over a series of workshops the PPI volunteers became expert in the field and made important design decisions.

In this paper we report the key features of the design of our RCT. We believe that screening for referable DR at intervals based on individual risk could be both safe and cost-effective compared with annual screening.

Funding and ethics approval

The ISDR RCT is part of a larger programme of applied research funded by a £2.1m grant from the National Institute for Health Research (NIHR) Programme Grants for Applied Research (PGfAR) programme – reference RG-PG-1210-12016. SPH is the Chief Investigator for the 7 workstream programmes and DMB the Principal Investigator for the RCT. The RCT is supported by the Clinical Trials Research Centre (CTRC) in Liverpool, which provides information systems support, developed electronic case report files and manage the data.

There are three trial oversight committees: a Trial Management Group, a Trial Steering Committee and the Independent Data and Safety Monitoring Committee. The composition and membership of the committees was approved by NIHR. The study protocol [37], patient information sheets and consent forms have received ethical approval from the National

For peer review only

Research Ethics Service Committee North West – Preston (reference: 14/NW/0034). Recruitment to the trial was supported by research staff from Clinical Research Network (CRN) North West Coast, LDESP staff and trained student volunteers.

Trial status

The trial opened to recruitment on the 12th November 2014. The first patient was randomised on the 19th November 2014. The trial was closed to enrolment on the 31 May 2016. The target of 4460 patients was reached. The Programme Grant completes on 30 April 2019.

Patient and Public Involvement

We have included members of our PPI Group in all stages of the design of the trial. Our PPI group consists of 7 individuals and has met at regular intervals throughout the lifetime of the trial. Individuals are active participants in the programme and trial steering committees, programme investigators committee and trial management committee. Individuals were involved in the concept and development of the research questions, design of the trial and the grant application. One patient is a co-investigator and was closely involved in designing the intervention including reviewing the potential burden. The group was involved in the choice of risk factors for consideration for the RCE and secondary outcome measures. They developed a set of patient centred outcomes, most notably adding visual impairment and need for treatment which they viewed as particularly important to patients. They considered the chance of missing disease if screen intervals were extended and after several structured sessions settled on a 2.5% risk as an acceptable limit. They were not involved in actual recruitment to the trial but they visited the screening centres and observed recruitment and gave constructive comments which improved the process.

For peer review only

Regarding dissemination of results, these will be sent to the participants' General Practitioners. Participants have also been advised to let the researcher know if they would like a summary of the results themselves.

Participants, interventions and outcomes

Trial design

The ISDR RCT had 1:1 allocation to a personalised risk based screening recall (6, 12 or 24 months) or annual screening (current routine care).

Objectives

The objective of the ISDR RCT is to evaluate the safety, acceptability and cost-effectiveness of personalised risk-based screening in a whole population setting. The main aim is to assess the relative safety of variable-interval screening as measured by attendance rate. We will test the hypothesis that the difference in attendance rates between the two pathways is within an acceptable equivalence margin.

Study setting

This single site trial is being conducted in all 7 screening clinics in the Liverpool Diabetic Eye Screening Programme (LDESP), which is part of the English National Diabetic Eye Screening Programme (NDESP).

Study population

People with diabetes registered with a GP whose postcode is in the city boundaries of Liverpool and attending for screening for DR were invited to enter the trial. The inclusion and exclusion criteria are given in Table 1.

For peer review only

Inclusion Criteria	Exclusion Criteria
adults, young people and children who are aged ≥12 years	under age 12 years
due to be offered an appointment for retinal screening during the recruitment period	
registered with a participating GP practice	are not registered with a participating GP practice
are included in the study data warehouse (have not opted out)	have opted out from the study data warehouse
have no retinopathy or have retinopathy and maculopathy less than the definition of screen positive diabetic retinopathy	have screen positive diabetic eye disease or significant other eye disease requiring referral to the HES
have gradeable digital retinal images in both eyes	are ineligible for screening for whatever reason, including having ungradable digital retinal images, which includes patients who have only one eye or an ungradable eye with no visual potential
give their informed consent for participation	do not give consent for participation in the RCT
are not involved in any trial investigating a treatment aiming at preventing or modifying the development of STDR	are involved in any trial investigating a treatment aiming at preventing or modifying the development of STDR

Table 1

The Schematic of the ISDR RCT Study Design is given in Figure 1.

Data flows

Data flows are shown in Figure 2. Data on the participant's retinopathy status feeds automatically from the screening software (OptoMize) into the data warehouse and then to the randomisation and RCE programmes on a daily basis. The ISDR data warehouse automatically populates the majority of the fields in the baseline and follow-up electronic CRFs (OpenClinica), including data that allowed randomisation to occur.

Primary endpoint

For peer review only

The primary outcome is the attendance rate for follow-up screening in the two arms of the study. Non-attendance is defined as failure to attend any screening appointments within 90 days of the expected follow-up date, irrespective of how many appointments they had been sent.

Secondary endpoints

- Number of cases of STDR detected
- Retinopathy level at screening
- Maculopathy level at screening
- Number of false positive screening episodes
- Number of screening appointments
- Number of diabetes assessment clinic appointments
- Number of other eye appointments for DR
- Visual acuity (logMAR)
- New visual impairment (≥ +0.50 and ≥ +0.50 logMAR)
- New visual impairment (≥ +0.50 and ≥ +0.50 logMAR) which in the opinion of an experienced clinician is due to DR
- Number of missed appointments to screening
- Patient acceptability measures (using a questionnaire designed for the RCT)
- Quality-adjusted life years (QALYs) estimated using EQ-5D-5L and Health Utilities Index Mark 3 (HUI3)
- Cost per QALY

For peer review only

Intervention, assessments and procedures

The ISDR data warehouse (DW) is a relational database which stores both the data and how the data are related. The Liverpool GPs gave permission for their PWD to be directly contacted to give permission for the data held in primary care relating to their diabetes to be transferred to the DW. The DW was established to collate and dynamically link individual risk factor data from GP practices, STDR outcome data from the DR assessment clinic at St Pauls Eye Unit and the LDESP screening software (OptoMize). This central data repository was used to develop the locally applicable risk calculation engine (RCE) described above using a continuous Markov model.

the (RCE) was developed using routinely collected primary and secondary care data from 11,806 PWD (46,525 screening episodes) in the LDESP. A detailed description of the RCE has already been published [33]. In brief, the final covariates for the RCE were selected using a 3-step decision process. A number of risk factors for developing STDR were identified in the published literature and in preliminary work in Liverpool. These potential covariates were reviewed by the clinical team and the PPI group and potential additional covariates were suggested. Potential covariates with a missingness rate of less than 20% in the data warehouse were identified. Then a statistical evaluation of the predictive value of each remaining covariate was undertaken. The covariates selected were disease state (current retinopathy levels in both eyes); age; duration of diabetes; glycated haemoglobin; systolic blood pressure; and total cholesterol. The results suggested that implementing personalised risk- based intervals would reduce the number of screen episodes by 30% [33].

Our PPI group defined the degree of risk (up to 2.5% risk of developing screen positive diabetic retinopathy) acceptable to people with diabetes, allowing assignment of individuals to screen intervals at the time points 6, 12 or 24 months. The choice of these intervals was

For peer review only

based on a review of the available literature, our data and consensus between the PPI and research teams. Following each negative screening outcome, individuals are assigned to the longest recall period up to 24 months at which their risk estimation would not exceed the 2.5% threshold.

Consent

Individuals received a Patient Information Leaflet with all the details of the trial with their screening appointment reminder letter. On attending the screening clinic they were approached by trained researchers to establish whether they wished to participate in the trial. If they expressed an interest the researcher took written informed consent and enrolled them into the trial. If an individual subsequently requested to be withdrawn from the trial they reverted to routine care. Reasons for, and level of, withdrawal were collected.

Randomisation

Randomisation could only be completed once the individual's retinal images had been graded, as it was only possible at this point to complete the inclusion and exclusion criteria. PWD who screened positive due to DR, other eye disease or ungradeable images were therefore consented but were not included in the trial.

Eligible PWD were randomly allocated to the personalised risk-based screening recall or the usual fixed annual interval. Participants were stratified by clinic and age (<16 and ≥16 years old). The screening clinics in Liverpool had different proportions of PWD in hard to reach groups, such as minority ethnic groups, attendance rates and social deprivation indices.

After randomisation the RCE was used to generate the individual's recall period in the personalised arm of the trial and the data was sent back to the screening software.

Study visits and assessments

For peer review only

At each follow-up visit, PWD randomised to the personalised risk-based screening arm have their risk re-calculated and the next recall interval determined accordingly.

Health economics

A health economics questionnaire, incorporating the health-related quality of life (QoL) questionnaires EQ-5D-5L [38] and Health Utilities Index Mark 3 (HUI3) [39] and a bespoke visit questionnaire [40], were completed by the first 868 eligible PWD enrolled into the study (a minimum of 700 PWD was required to provide sufficient data). The QoL questionnaires are repeated at every subsequent follow-up visit for those PWD who completed it at baseline.

For peer review

Trial Intervention diagram

				Follow-Up Schedule							
Procedures	*Screening*	*Baseline*	*Randomisation*	*6 months***	*12 months***	*18 months***	*24 months***	*30 months***	*36 months***	*Study Completion*	*Premature Discontinuation*
Signed Consent Form	*X*										
Visual Acuity(LogMar)		*X*		*X*	*X*	*X*	*X*	*X*	*X*		
Visit Questionnaire		*X*									
QoL Questionaire (HUI3 and EQ5D)*		*X*		*X*	*X*	*X*	*X*	*X*	*X*		
Assessment of Eligibility Criteria	*X*	*X*	*X*								
Randomisation			*X*								
Digital Imaging		*X*		*X*	*X*	*X*	*X*	*X*	*X*		
Acceptability Questionnaire									*X*		

*The EQ-5D-5L, HUI3 data will be collected on around 100-200 eligible PWD and repeated at each visit for those individual PWD. Data will be stratified to balance equal numbers of PWD in each arm and representation from all screening sites.

**PWD are randomised to 2 arms. In one arm individuals attend at 12 month intervals. In the other arm individuals attend at 6, 12 or 24 monthly intervals.

Sample size and planned analyses

The primary aim is to assess the relative safety of personalised risk-based interval screening as measured by equivalence in attendance rates. The analysis will test the hypothesis that the difference in attendance rates at the first follow-up between the two pathways is within the acceptable range δ=0.05. If the bounds of the 90% confidence interval for the difference in attendance rate are found to be within the confidence limits [$-\delta$,δ] (i.e. [-0.05, 0.05]) the results would support equivalence.

The estimated minimum number of patients required is 3940. With an expected loss to follow-up rate of 6% per year due to death and other exclusion from screening (note that non-attendance is the primary outcome and therefore is not factored here) the target for recruitment (randomised into the trial) was 4460 patients (4460*0.94*0.94=3940).

A secondary aim is to investigate whether personalised screening can be considered as non-inferior in detection of STDR when compared to annual screening. The STDR detection rate predicted for the usual care pathway during the two years follow-up is approximately 6%, based on data from the Liverpool DESP. The sample size required to address the first question (n=4460 patients randomised with 3940 patients retained after 2 years from baseline) will permit us to test for non-inferiority in STDR detection with a maximum allowable reduction of 1.5% of the personalised care pathway compared to standard care, with 5% significance level and power between 60-65%.

We will undertake sub-group analyses to assess differences in attendance rates between the two arms for the three different retinopathy groups (the risk groups will be defined based on the individual baseline estimated risk of developing STDR). A logistic mixed-effects model that takes into account the patient's covariate information over time (including HbA1c, systolic blood pressure, total cholesterol, disease duration, retinopathy level,

For peer review only

ethnicity, age, gender, smoking status, diabetes type), and screening clinic (clustered data) will be fitted with attendance (Yes/No) at the first screening visit as the outcome variable. The random effects of the model will account for the variability by screening clinic.

Using screening activity data stored in the ISDR DW and information collected from the visit questionnaire and EQ-5D-5L and HUI3 responses, we will estimate the cost per QALY within the study period associated with risk-based and annual screening from an NHS perspective and where possible incorporate a broader perspective. We will present bootstrapped incremental cost effectiveness ratios and cost effectiveness acceptability curves to characterise the uncertainty associated with our estimates.

We will apply sensitivity analyses to check the sensitivity of the results on the assumption that missing data are missing at random. Different scenarios for missing data mechanisms will be explored.

Conclusions

This paper describes the design of a RCT to evaluate the feasibility, safety, acceptability and cost-effectiveness of personalised variable-interval risk-based screening compared to fixed annual interval screening. Safety will be measured by the effect on attendance rates to screening, rates and severity of DR, visual outcomes and impact on general diabetes care. As far as we are aware this is the only study to investigate the impact of implementing personalised screening for diabetic retinopathy in an RCT.

For peer review only

Authors' contributions

DMB (Trial PI) and SPH (CI) conceived the trial questions and have been involved in all stages of the study design, and together with AEW (Trial Coordinator) and AW (ISDR Programme Manager) participated in writing the protocol, submission to the funding body and application to the ethics committee. SUH is a Senior Data Manager for the CTRC and DA is the information Systems Manager for the CTRC. Both contributed to writing the protocol and devised the electronic CRF system and data monitoring systems. MG-F is the lead statistician and co-investigator and CPC is the trial statistician. IMS is a leading UK statistician. All participated in writing the protocol and statistical aspects of the study. MMR is the data warehouse manager. MJ is the lead health economist and a co-investigator and CJS is the trial health economist. They designed the heath economic analysis. TM is a senior trials manager and a co-investigator. LH replaced AEW in the CTRC and has been involved in revising the protocol and preparing the manuscript with DMB and CJS. JK and JC are PPI members and contributed to the design of the RCE and the trial. All authors read and approved the final manuscript.

Legends

Figure 1: Schematic of the ISDR RCT trial design

Figure 2: Data Flows. In step 1 data for consented participants is requested from EMIS, passed to the Data Warehouse and cleaned prior to storage. In step 2 data is exchanged between the data warehouse and OptoMize (subjects whose risk needs to be calculated are sent to the data warehouse, subjects for whom their risk and therefore recall interval has been calculated is returned to OptoMize for appointment letter generation). In step 3 the participants risk is calculated (when all the covariates are available), if randomisation is

For peer review only

required they are then randomised. The data is then stored in the study database and the data warehouse. All processes in steps 1 and 2 involve identifiable data, the processes in step 3 all use pseudanonomised data for security reasons (the trial team with access to the trial database do not have a need to see raw identifiers). Step 1 occurs on a bi-monthy period, steps 2 & 3 occur on a daily basis. Under ideal conditions it takes three days for the data to pass through all parts of steps 1 and 2 – this is due to an airgap (manual transfer) at step 2 between the data warehouse and the NHS systems.

Table 1: Inclusion and exclusion criteria in the personalised variable-interval risk-based screening for diabetic retinopathy randomised controlled trial.

Competing interests

The authors report no competing interests

Funding statement

This paper presents independent research funded by the National Institute for Health Research (NIHR) under the Programme Grants for Applied Research programme (RP-PG-1210-12016). The views expressed are those of the authors and not necessarily those of the NHS, the NIHR or the Department of Health.

Data sharing statement

The formal protocol is published at 2014 DOI: 10.6084/m9.figshare.5053336.v1

Trial sponsors

University of Liverpool Research Support Office,

1st floor Duncan Building,

Daulby Street,

Liverpool L69 3GA.

For peer review only

Tel: 0151 706 3702

Research and Innovation Department

Royal Liverpool and Broadgreen University Hospitals Trust

Prescot Street

Liverpool

L7 8XP

0151 706 2000

University of Liverpool: UoL000994

Research and Innovation Department

Royal Liverpool and Broadgreen University Hospitals Trust

Prescot Street

Liverpool

L7 8XP

0151 706 2000

Trust RD&I: 4660

ISDR Study Group

Simon P Harding (Study Group Chair), Deborah M Broadbent (Trial PI), Anthony C Fisher, Mark Gabbay, Marta García-Fiñana, Marilyn James, Tracy Moitt, John R Roberts, Daniel Seddon, Irene M Stratton, Paula Williamson, Duncan Appelbe, Andy Ovens, Lola Howard, Susan U Howlin, Ayesh Alshukri, Abigail E Williams, Christopher P Cheyne, Antonio Eleuteri, Christopher Grierson, Mehrdad Mobayen-Rahni, Christopher J Sampson, David Szmyt, Clare Thetford, Maria Pilar Vazquez Arango, Amu Wang, Helen Cooper, John Collins, John Kelly, Nathalie Massat, Gideon Smith, Vineeth Kumar, Chris Rogers, Julia West, Naveed Younis,

For peer review only

Alison Rowlands, Peter Lees, Sandra Lees, Emily Doncaster, Betty Williams, Ticiana Criddle, Stephanie Perrett.

Acknowledgments

We would like to acknowledge the outstanding attention to detail from the ISDR administrative team and the CTRC. We would like to thank the North West Coast CRN and our other trained researchers for their invaluable help in recruiting to target. We would also like to thank the LDESP for their support and assistance. We would particularly like to thank our PPI members for their invaluable advice and enthusiastic support for the study.

Christopher J Sampson is currently affiliated to the Office of Health Economics, Southside, 7th Floor, 105 Victoria Street, London SW1E 6QT

References

1. Klein R, Klein BE, Moss SE, Davis MD, DeMets DL. The Wisconsin epidemiologic study of diabetic retinopathy. II. Prevalence and risk of diabetic retinopathy when age at diagnosis is less than 30 years. *Arch Ophthalmol* 1984, 102:520-526.

2. Klein R, Klein BE, Moss SE, Davis MD, DeMets DL. The Wisconsin epidemiologic study of diabetic retinopathy. III. Prevalence and risk of diabetic retinopathy when age at diagnosis is 30 or more years. *Arch Ophthalmol* 1984, 102:527-532

3. Liew, G, Michaelides, M, Bunce, C. A comparison of the causes of blindness certifications in England and Wales in working age adults (16 to 64 years), 1999 to 2000 with 2009 to 2010. *BMJ Open* 2014, 12 February 2014

4. PHE bulletin 2016 https://www.gov.uk/government/uploads/system/uploads/attachment_data/file/556910/PHE_Bulletin_September_2016.pdf Last accessed 03.01.18

For peer review only

5. NHS diabetic eye screening programme. Diabetic eye screening: commission and provide. https://www.gov.uk/government/collections/diabetic-eye-screening-commission-and-provide Last accessed 03.01.18

6. UK National Screening Committee. The UK NSC recommendation on Diabetic Retinopathy screening in adults. January 2016. Available at http://legacy.screening.nhs.uk/diabeticretinopathy. Last accessed 03.01.18

7. Scanlon PH, Aldington, SJ, Leal J, Luengo-Fernandez R, Oke J, Sivaprasad S et al. Development of a cost-effectiveness model for optimisation of the screening interval in diabetic retinopathy screening. *Health Technol Assess* 2015;19:1-116

8. Taylor-Phillips S, Mistry H, Leslie R, Todkill D, Tsertd vadze A, Connock M et al. Extending the diabetic retinopathy screening interval beyond 1 year: systematic review. *Br J Ophthalmol* 2016;100:105-114

9. Leese GP, Stratton IM, Land M, Bachmann MO, Jones C, Scanlon P et al. Progressio of diabetes retinal status within community screening programs and potential implications for screening intervals. *Diabetes Care* 2015;38:488-494

10. Davies R, Sullivan P, Canning C. Simulation of diabetic eye disease to compare screening policies. *Br J Ophthalmol* 1996, 80:945-950.

11. Younis N, Broadbent DM, Vora JP, Harding SP. Incidence of sight-threatening retinopathy in patients with Type 2 diabetes in the Liverpool Diabetic Eye Study: a cohort study. *Lancet* 2003, 361:195-200

12. Younis N, Broadbent DM, Harding SP, Vora JP. Incidence of sight-threatening retinopathy in Type 1 diabetes in a systematic screening programme. *Diabet Med* 2003, 20:758-765

For peer review only

13. Stefansson E, Bek T, Porta M, Larsen N, Kristinsson JK, Agardh E. Screening and prevention of diabetic blindness. *Acta Ophthalmol Scand* 2000;78:374-385

14. Agardh E, Tabayat-Khani P. Adopting 3-year screening intervals for sight-threatening retinal vascular lesions in Type 2 diabetic subjects without retinopathy. *Diab Care* 2011;34:1-2

15. Javitt JC, Aiello LP: Cost-Effectiveness of Detecting and Treating Diabetic Retinopathy. *Annals of Internal Medicine* 1996, 124:164-169.

16. Jones S, Edwards RT. Diabetic retinopathy screening: a systematic review of the economic evidence. *Diabet Med* 2010, 27(3):249–56. DOI: 10.1111/j.1464-5491.2009.02870.x

17. James M, Turner DA, Broadbent DM, Vora J, Harding SP. Cost effectiveness analysis of screening for sight threatening diabetic eye disease. *BMJ* 2000, 320(7250):1627–31

18. Stratton IM, Aldington SJ, Taylor DJ, Adler AI, Scanlon PH. A simple risk stratification for time to development of sight-threatening diabetic retinopathy. *Diabetes Care* 2013 Mar; 36(3):580-5. DOI: 10.2337/dc12-0625

19. Scanlon PH, Stratton IM, Histed M, Chave SJ, Aldington SJ. The influence of background diabetic retinopathy in the second eye on rates of progression of diabetic retinopathy between 2005 and 2010. *Acta Ophthalmol* 2013 Aug; 91(5):e335-9. DOI: 10.1111/aos.12074

20. Taylor-Phillips S, Mistry H, Leslie R et al. Extending the diabetic retinopathy screening interval beyond 1 year: systematic review. http://dx.doi.org/10.1136/bjophthalmol-2014-305938

For peer review only

21. The Diabetes Control and Complications Trial Research Group. The effect of intensive treatment of diabetes on the development and progression of long-term complications in insulin-dependent diabetes mellitus. *N Engl J Med* 1993, 329:977-986

22. Davis MD, Fisher MR, Gangnon RE, Barton F, Aiello LM, Chew EY, Ferris FL, 3rd, Knatterud GL. Risk factors for high-risk proliferative diabetic retinopathy and severe visual loss: Early Treatment Diabetic Retinopathy Study Report #18. *Invest Ophthalmol Vis Sci* 1998, 39:233-252

23. Marshall G, Garg SK, Jackson WE, Holmes DL, Chase HP. Factors influencing the onset and progression of diabetic retinopathy in subjects with insulin-dependent diabetes mellitus. *Ophthalmology* 1993, 100:1133-1139

24. Stratton IM, Kohner EM, Aldington SJ, Turner RC, Holman RR, Manley SE, Matthews DR: UKPDS 50. Risk factors for incidence and progression of retinopathy in Type II diabetes over 6 years from diagnosis. *Diabetologia* 2001, 44:156-163

25. Agardh CD, Agardh E, Torffvit O. The association between retinopathy, nephropathy, cardiovascular disease and long-term metabolic control in Type 1 diabetes mellitus: a 5 year follow-up study of 442 adult patients in routine care. *Diabetes Res Clin Pract* 1997, 35:113-121

26. Vijan S, Hofer TP, Hayward RA. Estimated Benefits of Glycemic Control in Microvascular Complications in Type 2 Diabetes. *Annals of Internal Medicine* 1997, 127:788-795

27. UK Prospective Diabetes Study Group. Tight blood pressure control and risk of macrovascular and microvascular complications in Type 2 diabetes: UKPDS 38. . *BMJ* 1998, 317:703-713

For peer review only

28. Gallego PH, Craig ME, Hing S, Donaghue KC. Role of blood pressure in development of early retinopathy in adolescents with Type 1 diabetes: prospective cohort study. *BMJ* 2008, 337:a918

29. Chaturvedi N, Sjolie AK, Stephenson JM, Abrahamian H, Keipes M, Castellarin A, Rogulja-Pepeonik Z, Fuller JH. Effect of lisinopril on progression of retinopathy in normotensive people with Type 1 diabetes. The EUCLID Study Group. EURODIAB Controlled Trial of Lisinopril in Insulin-Dependent Diabetes Mellitus. *Lancet* 1998, 351:28-31

30. Keech A, Simes RJ, Barter P, Best J, Scott R, Taskinen MR, Forder P, Pillai A, Davis T, Glasziou P, et al. Effects of long-term fenofibrate therapy on cardiovascular events in 9795 people with Type 2 diabetes mellitus (the FIELD study): randomised controlled trial. *Lancet* 2005, 366:1849-1861

31. Klein R, Klein BEK, Moss SE, Cruickshanks KJ. The Wisconsin epidemiologic study of diabetic retinopathy: XVII: The 14-year incidence and progression of diabetic retinopathy and associated risk factors in Type 1 diabetes. *Ophthalmology* 1998, 105:1801-1815

32. Sampson CJ, James M, Broadbent DM, Harding SP. Stratifying the NHS Diabetic Eye Screening Programme: into the unknown? *Diabet Med* 2016, 33(12): 1612–4. DOI: 10.1111/dme.13192

33. Eleuteri A, Fisher AC, Broadbent DM, García-Fiñana M, Cheyne CP, Wang A, Stratton IM, Gabbay M, Seddon D, Harding SP for the ISDR Study Group. Individualised variable interval risk-based screening for sight threatening diabetic retinopathy – the Liverpool Risk Calculation Engine. *Diabetologia* 2017, 60(11): 2174-2182

34. Aspelund T, Porisdottir O, Olafsdottir E, Gudmundsdottir A, Einarsdottir AB, Mehlsen J, Einarsson S, Palsson O, Bek T, Stefansson E. Individual risk assessment and information

For peer review only

technology to optimise screening frequency for diabetic retinopathy. *Diabetologia* 2011, DOI: 10.1007/s00125-011-2257-7

35. van der Heijden AA, Walraven I, van't Riet E et al. Validation of a model to estimate personalised screening frequency to monitor diabetic retinopathy. *Diabetologia* 2014, 57(7):1332-1338

36. Yeo ST, Edwards RT, Luzio SD, Charles JM, Thomas RL, Peters JM, et al. Diabetic retinopathy screening: perspectives of people with diabetes, screening intervals and costs of attending screening. *Diabet Med* 2012, 29(7):878–85. DOI: 10.1111/j.1464-5491.2012.03637.x

37. Broadbent DM, Bennett A, Moitt T, Howlin S, James M, Sampson C, et al. Introducing personalised risk based intervals in screening for diabetic retinopathy: development, implementation and assessment of safety, cost-effectiveness and patient experience. Workstream E: Randomised trial comparing standard and test screening intervals protocols. 2014 DOI: 10.6084/m9.figshare.5053336.v1

38. Herdman M, Gudex C, Lloyd A, Janssen M, Kind P, Parkin D, et al. Development and preliminary testing of the new five-level version of EQ-5D (EQ-5D-5L). *Qual Life Res* 2011, 20(10):1727–36. DOI: 10.1007/s11136-011-9903-x

39. Feeny D, Furlong W, Boyle M, Torrance GW. Multi-attribute health status classification systems. Health Utilities Index. *Pharmacoeconomics* 1995, 7(6):490–502. DOI: 10.2165/00019053-199507060-00004

40. James M, Sampson C. ISDR Visit Questionnaire. Database of Instruments for Resource Use Measurement. 2015. DOI: Available from: http://dirum.org/instruments/details/90 DOI: 10.6084/m9.figshare.1301022.v1

For peer review only

For peer review only

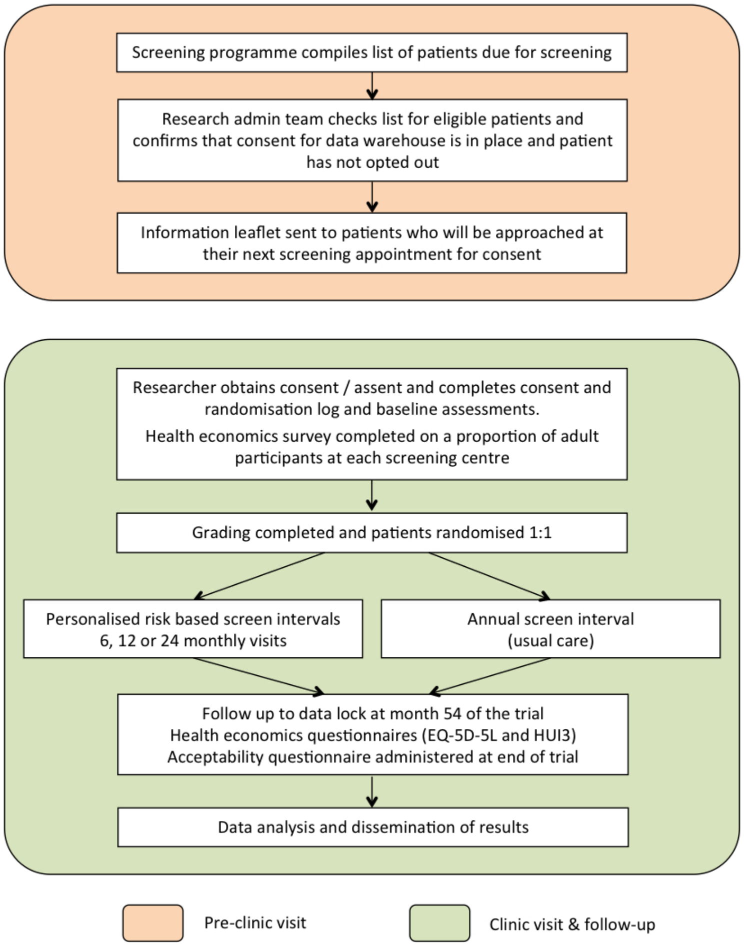

Figure 1: Schematic of the ISDR RCT trial design

352x470mm (72 x 72 DPI)

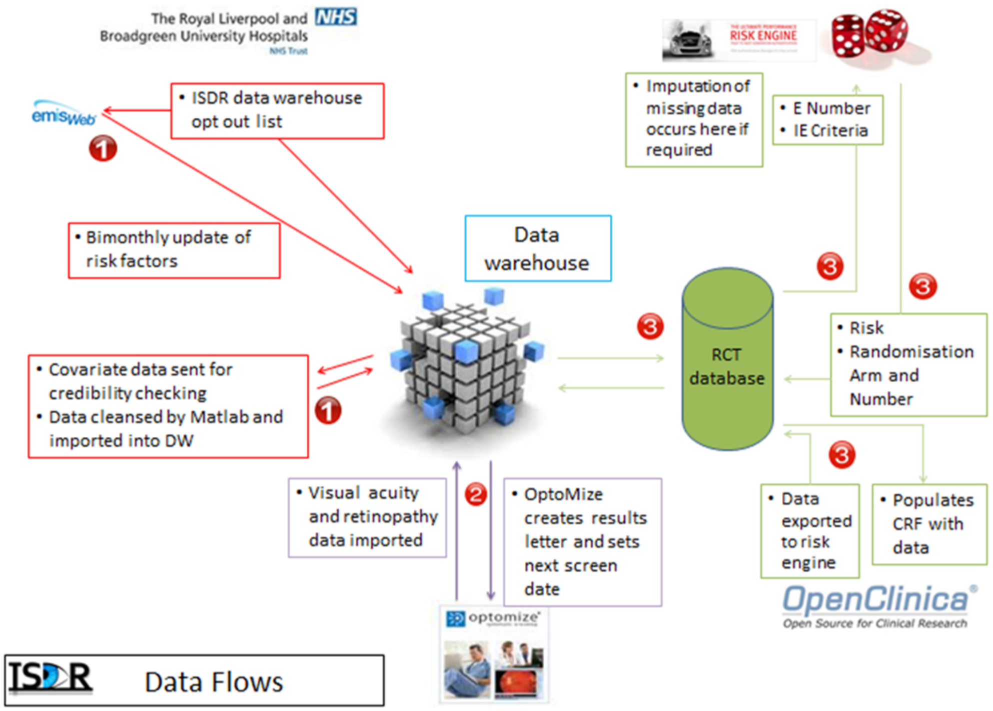

Figure 2: Data Flows. In step 1 data for consented participants is requested from EMIS, passed to the Data Warehouse and cleaned prior to storage. In step 2 data is exchanged between the data warehouse and OptoMize (subjects whose risk needs to be calculated are sent to the data warehouse, subjects for whom their risk and therefore recall interval has been calculated is returned to OptoMize for appointment letter generation). In step 3 the participants risk is calculated (when all the covariates are available), if randomisation is required they are then randomised. The data is then stored in the study database and the data warehouse. All processes in steps 1 and 2 involve identifiable data, the processes in step 3 all use pseudanonomised data for security reasons (the trial team with access to the trial database do not have a need to see raw identifiers). Step 1 occurs on a bi-monthy period, steps 2 & 3 occur on a daily basis. Under ideal conditions it takes three days for the data to pass through all parts of steps 1 and 2 – this is due to an airgap (manual transfer) at step 2 between the data warehouse and the NHS systems.

90x90mm (300 x 300 DPI)

STANDARD PROTOCOL ITEMS: RECOMMENDATIONS FOR INTERVENTIONAL TRIALS

SPIRIT 2013 Checklist: Recommended items to address in a clinical trial protocol and related documents*

Section/item	Item No	Description	Addressed on page number
**Administrative information**			
Title	1	Descriptive title identifying the study design, population, interventions, and, if applicable, trial acronym	___3_________
Trial registration	2a	Trial identifier and registry name. If not yet registered, name of intended registry	___6_________
	2b	All items from the World Health Organization Trial Registration Data Set	__NA**________
Protocol version	3	Date and version identifier	__NA**________
Funding	4	Sources and types of financial, material, and other support	___11________
Roles and responsibilities	5a	Names, affiliations, and roles of protocol contributors	____3________
	5b	Name and contact information for the trial sponsor	____22/23___
	5c	Role of study sponsor and funders, if any, in study design; collection, management, analysis, and interpretation of data; writing of the report; and the decision to submit the report for publication, including whether they will have ultimate authority over any of these activities	____22_______
	5d	Composition, roles, and responsibilities of the coordinating centre, steering committee, endpoint adjudication committee, data management team, and other individuals or groups overseeing the trial, if applicable (see Item 21a for data monitoring committee)	____11________

For peer review only

**Introduction**			
Background and rationale	6a	Description of research question and justification for undertaking the trial, including summary of relevant studies (published and unpublished) examining benefits and harms for each intervention	____7-11_____
	6b	Explanation for choice of comparators	____7-11_____
Objectives	7	Specific objectives or hypotheses	_____13______
Trial design	8	Description of trial design including type of trial (eg, parallel group, crossover, factorial, single group), allocation ratio, and framework (eg, superiority, equivalence, noninferiority, exploratory)	_____13______
**Methods: Participants, interventions, and outcomes**			
Study setting	9	Description of study settings (eg, community clinic, academic hospital) and list of countries where data will be collected. Reference to where list of study sites can be obtained	_____13______
Eligibility criteria	10	Inclusion and exclusion criteria for participants. If applicable, eligibility criteria for study centres and individuals who will perform the interventions (eg, surgeons, psychotherapists)	_Table 1_____
Interventions	11a	Interventions for each group with sufficient detail to allow replication, including how and when they will be administered	____13,16/17__
	11b	Criteria for discontinuing or modifying allocated interventions for a given trial participant (eg, drug dose change in response to harms, participant request, or improving/worsening disease)	____17 _____
	11c	Strategies to improve adherence to intervention protocols, and any procedures for monitoring adherence (eg, drug tablet return, laboratory tests)	___NA_______
	11d	Relevant concomitant care and interventions that are permitted or prohibited during the trial	___NA_______
Outcomes	12	Primary, secondary, and other outcomes, including the specific measurement variable (eg, systolic blood pressure), analysis metric (eg, change from baseline, final value, time to event), method of aggregation (eg, median, proportion), and time point for each outcome. Explanation of the clinical relevance of chosen efficacy and harm outcomes is strongly recommended	____15_______
Participant timeline	13	Time schedule of enrolment, interventions (including any run-ins and washouts), assessments, and visits for participants. A schematic diagram is highly recommended (see Figure)	___Fig 1_____

For peer review only - http://bmjopen.bmj.com/site/about/guidelines.xhtml

Sample size	14	Estimated number of participants needed to achieve study objectives and how it was determined, including clinical and statistical assumptions supporting any sample size calculations	____19-20____
Recruitment	15	Strategies for achieving adequate participant enrolment to reach target sample size	_____achieved

Methods: Assignment of interventions (for controlled trials)

Allocation:

Sequence generation	16a	Method of generating the allocation sequence (eg, computer-generated random numbers), and list of any factors for stratification. To reduce predictability of a random sequence, details of any planned restriction (eg, blocking) should be provided in a separate document that is unavailable to those who enrol participants or assign interventions	_____17____
Allocation concealment mechanism	16b	Mechanism of implementing the allocation sequence (eg, central telephone; sequentially numbered, opaque, sealed envelopes), describing any steps to conceal the sequence until interventions are assigned	_____17____
Implementation	16c	Who will generate the allocation sequence, who will enrol participants, and who will assign participants to interventions	_____17____
Blinding (masking)	17a	Who will be blinded after assignment to interventions (eg, trial participants, care providers, outcome assessors, data analysts), and how	____NA______
	17b	If blinded, circumstances under which unblinding is permissible, and procedure for revealing a participant's allocated intervention during the trial	____NA______

Methods: Data collection, management, and analysis

Data collection methods	18a	Plans for assessment and collection of outcome, baseline, and other trial data, including any related processes to promote data quality (eg, duplicate measurements, training of assessors) and a description of study instruments (eg, questionnaires, laboratory tests) along with their reliability and validity, if known. Reference to where data collection forms can be found, if not in the protocol	______11_____
	18b	Plans to promote participant retention and complete follow-up, including list of any outcome data to be collected for participants who discontinue or deviate from intervention protocols	____NA**_____

Data management	19	Plans for data entry, coding, security, and storage, including any related processes to promote data quality (eg, double data entry; range checks for data values). Reference to where details of data management procedures can be found, if not in the protocol	_____11______
Statistical methods	20a	Statistical methods for analysing primary and secondary outcomes. Reference to where other details of the statistical analysis plan can be found, if not in the protocol	______19-20___
	20b	Methods for any additional analyses (eg, subgroup and adjusted analyses)	______19-20___
	20c	Definition of analysis population relating to protocol non-adherence (eg, as randomised analysis), and any statistical methods to handle missing data (eg, multiple imputation)	______19-20___
**Methods: Monitoring**			
Data monitoring	21a	Composition of data monitoring committee (DMC); summary of its role and reporting structure; statement of whether it is independent from the sponsor and competing interests; and reference to where further details about its charter can be found, if not in the protocol. Alternatively, an explanation of why a DMC is not needed	______11**____
	21b	Description of any interim analyses and stopping guidelines, including who will have access to these interim results and make the final decision to terminate the trial	___ NA**_____
Harms	22	Plans for collecting, assessing, reporting, and managing solicited and spontaneously reported adverse events and other unintended effects of trial interventions or trial conduct	____NA**______
Auditing	23	Frequency and procedures for auditing trial conduct, if any, and whether the process will be independent from investigators and the sponsor	_____11______
**Ethics and dissemination**			
Research ethics approval	24	Plans for seeking research ethics committee/institutional review board (REC/IRB) approval	_____11_____
Protocol amendments	25	Plans for communicating important protocol modifications (eg, changes to eligibility criteria, outcomes, analyses) to relevant parties (eg, investigators, REC/IRBs, trial participants, trial registries, journals, regulators)	_____NA_____

For peer review only - http://bmjopen.bmj.com/site/about/guidelines.xhtml

Consent or assent	26a	Who will obtain informed consent or assent from potential trial participants or authorised surrogates, and how (see Item 32)	_____17_______
	26b	Additional consent provisions for collection and use of participant data and biological specimens in ancillary studies, if applicable	_____NA_______
Confidentiality	27	How personal information about potential and enrolled participants will be collected, shared, and maintained in order to protect confidentiality before, during, and after the trial	_____11**_______
Declaration of interests	28	Financial and other competing interests for principal investigators for the overall trial and each study site	_____22______
Access to data	29	Statement of who will have access to the final trial dataset, and disclosure of contractual agreements that limit such access for investigators	______NA_______
Ancillary and post-trial care	30	Provisions, if any, for ancillary and post-trial care, and for compensation to those who suffer harm from trial participation	______NA_____
Dissemination policy	31a	Plans for investigators and sponsor to communicate trial results to participants, healthcare professionals, the public, and other relevant groups (eg, via publication, reporting in results databases, or other data sharing arrangements), including any publication restrictions	______13______
	31b	Authorship eligibility guidelines and any intended use of professional writers	____ NA**______
	31c	Plans, if any, for granting public access to the full protocol, participant-level dataset, and statistical code	______NA______
**Appendices**			
Informed consent materials	32	Model consent form and other related documentation given to participants and authorised surrogates	____NA________
Biological specimens	33	Plans for collection, laboratory evaluation, and storage of biological specimens for genetic or molecular analysis in the current trial and for future use in ancillary studies, if applicable	____NA________

*It is strongly recommended that this checklist be read in conjunction with the SPIRIT 2013 Explanation & Elaboration for important clarification on the items. Amendments to the protocol should be tracked and dated. The SPIRIT checklist is copyrighted by the SPIRIT Group under the Creative Commons "Attribution-NonCommercial-NoDerivs 3.0 Unported" license.

For peer review only - http://bmjopen.bmj.com/site/about/guidelines.xhtml

** This is a Rationale paper rather than a published protocol. Where asterisked these details are available in the Protocol at 2014 DOI: 10.6084/m9.figshare.5053336.v1

For peer review only

For peer review only - http://bmjopen.bmj.com/site/about/guidelines.xhtml

BMJ Open

Individualised Screening for Diabetic Retinopathy: the ISDR study. Rationale, design and methodology for a randomised controlled trial comparing annual and personalised variable-interval risk-based screening

Journal:	*BMJ Open*
Manuscript ID	bmjopen-2018-025788.R2
Article Type:	Protocol
Date Submitted by the Author:	13-Feb-2019
Complete List of Authors:	Broadbent, Deborah; Royal Liverpool University Hospital, St Paul's Eye Unit;<br>Sampson, Christopher; Office of Health Economics, ;<br>Wang, Amu; University of Liverpool , Eye and Vision Science<br>Howard, Lola; University of Liverpool, Department of Biostatistics, ;<br>Williams, Abigail; University of Liverpool, Biostatistics<br>Howlin, Susan; Liverpool John Moores University School of Humanities and Social Science, Biostatistics<br>Appelbe, Duncan; University of Liverpool, Clinical Trials Research Centre<br>Moitt, Tracy; University of Liverpool Department of Biostatistics,<br>Cheyne, C; University of Liverpool, Biostatistics<br>Rahni, Mehrdad; Department of Eye and Vision Science, University of Liverpool, Institute of Ageing and Chronic Disease<br>Kelly, John; Royal Liverpool University Hospital, St Paul's Eye Unit<br>Collins, John; Royal Liverpool University Hospital, Department of Eye andVision Science<br>García-Fiñana, Marta; University of Liverpool, Biostatistics<br>Stratton, Irene; Gloucester Hospitals NHSFT, Gloucestershire Retinopathy research Group; home,<br>James, Marilyn; University of Nottingham, School of Medicine<br>Harding, Simon; Department of Eye and Vision Science, University of Liverpool, Institute of Ageing and Chronic Disease
**Primary Subject Heading**:	Ophthalmology
Secondary Subject Heading:	Diabetes and endocrinology, Health economics, Health services research, Public health, Patient-centred medicine
Keywords:	Diabetic retinopathy < DIABETES & ENDOCRINOLOGY, General diabetes < DIABETES & ENDOCRINOLOGY, Health economics < HEALTH SERVICES ADMINISTRATION & MANAGEMENT, Health policy < HEALTH SERVICES ADMINISTRATION & MANAGEMENT, Medical ophthalmology < OPHTHALMOLOGY, Medical retina < OPHTHALMOLOGY

For peer review only - http://bmjopen.bmj.com/site/about/guidelines.xhtml

For peer review only - http://bmjopen.bmj.com/site/about/guidelines.xhtml

Individualised Screening for Diabetic Retinopathy: the ISDR study. Rationale, design and methodology for a randomised controlled trial comparing annual and personalised variable-interval risk-based screening.

Authors

Deborah M Broadbent,[1,2*] Christopher J Sampson,[3] Amu Wang, [1] Lola Howard,[4] Abigail E Williams, [4], Susan U Howlin,[4] Duncan Appelbe,[4] Tracy Moitt,[4] Christopher P Cheyne,[4,5] Mehrdad Mobayen Rahni,[1] John Kelly,[6] John Collins,[6] Marta García-Fiñana,[4,5] Irene M Stratton,[7] Marilyn James,[3] Simon P Harding[1,2] for the ISDR Study Group.

Affiliations

1. Department of Eye and Vision Science, Institute of Ageing and Chronic Disease, University of Liverpool, UK
2. St. Pauls Eye Unit, Royal Liverpool University Hospital, UK
3. Division of Rehabilitation and Ageing, School of Medicine, University of Nottingham, UK
4. Department of Biostatistics, Clinical Trials Research Centre, University of Liverpool, UK
5. Department of Biostatistics, University of Liverpool, UK
6. ISDR Patient and Public Involvement Group
7. Gloucestershire Retinal Research Group, Cheltenham General Hospital, Cheltenham, UK

Correspondence:

Dr Deborah M Broadbent

Honorary Senior Lecturer,

Department of Eye and Vision Science,

For peer review only

3rd Floor University Clinical Departments

Royal Liverpool University Hospital

Prescot Street

Liverpool L7 8XP

Tel +44 151 706 2408

Fax +44 151 706 2409

deborah.broadbent@rlbuht.nhs.uk

Key words

Diabetic retinopathy, risk calculation engine, risk-based screening, cost-effectiveness, personalised medicine

Word count 3538

For peer review only

Abstract

Introduction

Currently all people with diabetes (PWD) aged 12 years and over in the UK are invited for screening for diabetic retinopathy (DR) annually. Resources are not increasing despite a 5% increase in the numbers of PWD nationwide each year. We describe the rationale, design and methodology for a randomised controlled trial (RCT) evaluating the safety, acceptability and cost effectiveness of personalised variable-interval risk-based screening for DR. This is the first randomised trial of personalised screening for DR and the largest ophthalmic RCT in the UK.

Methods and analysis

PWD attending 7 screening clinics in the Liverpool Diabetic Eye Screening Programme (LDESP) were recruited into a single site RCT with a 1:1 allocation to personalised variable-interval risk-based or annual screening intervals.

A Risk Calculation Engine developed for the trial estimates the probability that an individual will develop referable disease (screen positive DR) within the next 6, 12 or 24 months using demographic, retinopathy and systemic risk factor data from primary care and screening programme records. Dynamic, secure, real-time data connections have been developed.

The primary outcome is attendance for follow-up screening. We will test for equivalence in attendance rates between the two arms. Secondary outcomes are rates and severity of DR, visual outcomes, cost effectiveness and health-related quality of life.

The required sample size was 4460 PWD. Recruitment is complete and the trial is in follow-up.

Ethics and dissemination

For peer review only

Ethical approval was obtained from NRES Committee North West – Preston, reference 14/NW/0034. Results will be presented at international meetings and published in peer-reviewed journals. This pragmatic RCT will inform screening policy in the UK and elsewhere.

Strengths and limitations of the study

- Our study addresses one of the primary current issues in the field of screening for diabetic retinopathy, the safety of extending the interval between episodes.
- Our study has significant novelty applying a personalised approach to a whole population intervention, using routinely collected data from primary and secondary care, a risk calculation engine that allows generalisation, and evaluated in the first RCT conducted on screening for diabetic retinopathy,
- Strengths include the substantial involvement of people with diabetes in the design and implementation, large numbers of participants for tight confidence limits, pragmatic solutions for variable quality routine NHS data and the independent analysis by an accredited clinical trials unit.
- The risk calculation engine has been internally validated but will require further external validation before implementation could be considered elsewhere.
- The trial only recruits people currently attending screening and does not identify the impact for people who currently do not attend.

Trial registration number: ISRCTN 87561257

For peer review

Introduction

Background

Diabetes mellitus (DM) is a life-long condition associated with the development of various macro- and microvascular complications, including diabetic retinopathy (DR) and maculopathy (a subgroup within DR). These are progressive conditions of the retina and macula, which can lead to visual impairment and blindness. There are an estimated 3.8 million people with diabetes (PWD) aged 16 years and over in England [1], of whom almost 1 million are undiagnosed. Nearly all people with type 1 diabetes [2] and over 60% with type 2 diabetes [3] will develop some degree of DR after 20 years of having diabetes.

DR affects people of all ages and is the commonest cause of blindness in people of working age in most developed countries worldwide. In the UK this position has changed. Liew et al reviewed the causes of blindness certifications in working age adults in England and Wales in 2009-2010 and reported that DR had dropped to the second most common cause compared with ten years earlier [4]. Tighter control of glycaemia and hypertension will have contributed to this but it is probable that screening for DR has also played a key role.

The early stages of DR (background and mild pre-proliferative) are asymptomatic and do not require any treatment. Sight threatening diabetic retinopathy (STDR - an umbrella term that encompasses sight threatening levels of retinopathy and maculopathy) requires close monitoring by an ophthalmologist, and sometimes treatment, to prevent visual impairment (VI).

Treatment (by laser photocoagulation or intravitreal injections) aims to stop progression and stabilise retinopathy. Treatment cannot always reverse the process once vision is lost. Screening is therefore recommended to identify STDR at the optimal time-point for treatment. Previous studies have shown that screening for DR is a highly cost-effective

For peer review only

intervention [5, 6]: the higher the take-up rates for screening, the higher the cost effectiveness [7].

Annual systematic screening for DR for all PWD aged twelve years or older was introduced across the UK by 2007. A one-year interval between screening invitations (screen interval) was based on expert opinion, rather than direct evidence. In 2016 the UK National Screening Committee (NSC) recommended that PWD at low risk of sight loss could be screened every two years [8] but this has not yet been adopted. This stratified approach was based on evidence from a large observational study in one English programme and a cost effectiveness analysis [9]. The findings were validated in the Welsh, Scottish and NI programmes and 4 more English programmes. Stratified screening is due to be implemented in Scotland.

Available evidence shows that many people at low risk of developing referable DR between annual screening appointments could safely be screened less often [10], whilst others are at high risk and might benefit from more frequent screening [11]. In 1996 Davies et al used simulation modelling of published data and concluded that biennial screening could be considered where patient compliance and screening sensitivities were both high [12]. Data on 10-year incidence from the Liverpool Diabetic Eye Study (LDES) - for a population of people with type 2 DM and enrolled in a systematic screening programme - suggested that a 3-yearly screening interval could be adopted for PWD with no retinopathy at baseline, but yearly or more frequent screening was recommended for people with higher grades of retinopathy or insulin use [13]. Similar results were shown for type 1 DM [14]. In Sweden, biennial screen intervals have been used for some time for subjects without retinopathy [15]. A study carried out in Malmo prospectively followed people with type 2 DM and no retinopathy and concluded that it appeared safe to adopt 3-year intervals [16] as suggested

For peer review only

by the Liverpool group. However, this group of PWD were compliant (only 9% did not attend for follow-up), had a short duration of diabetes (6 ± 6 years) and good control (HbA1c 6.4 ± 1.5% at baseline and 6.3 ± 1.3% at 3 year follow up).

Two studies have looked at stratified screening and shown that the risk of progression to STDR is significantly higher for those with background DR (BDR) in both eyes than those with BDR in only one or in neither eye [17, 18]. The first of these two studies suggested that combining the results from 2 consecutive years of photographic screening enabled estimation of the risk of future development of STDR. People with no DR on two consecutive visits were deemed to be low risk.

So why hasn't an extended interval in screening for STDR been more widely adopted? There has been considerable concern about the safety of extending screen intervals and, to date, no RCT has reported [19]. The experience of Sweden and Scotland is reassuring but the populations are much smaller and generally better engaged. If people receive the impression that they are at low risk they may disengage with other aspects of diabetes care. Despite encouraging findings from our group in 2003, in the absence of the required safety data it appears unwise to recommend 2 or 3-year intervals for people with diabetes at low risk without further evidence.

Rationale for a RCT

With this in mind we designed a RCT to investigate the safety of extending screening intervals in low risk PWD. We included the emerging theories and technologies of risk prediction and personalisation to develop a variable-interval risk-based screening approach and incorporated an economic evaluation.

In addition to severity of retinopathy, the risk of development and progression of DR to a level that requires treatment is related to age, gender [1,2], duration of diabetes, HbA1c

For peer review only

levels [20-25], blood pressure [26-28], lipid levels [29] and proteinuria [30]. If the contribution of each risk factor to overall risk (as well as the overall contribution in combination) could be calculated, and data on each risk factor were available for individuals, individual risks could be estimated and screening frequencies could be tailored to the level of personal risk [31].

We developed a risk calculation engine (RCE) using a longitudinal dataset from our local diabetic population to predict the risk of developing STDR, and have reported confident estimates of the risk of having STDR [32]. In Iceland a similar 'risk engine' has also been developed to estimate the risk of development of STDR, but based on modelling of historic epidemiological data on a limited number of risk factors for DR [33]. In the Netherlands a screening model based on patients' risk has been validated in 3319 people with type 2 diabetes as part of the Hoorn Study [34]. We embedded a patient and public involvement (PPI) group in the development of the RCE and subsequent design of the trial. There has been scant research into people's understanding of screening and their views about introducing variable screening intervals. Yeo et al (2012) reported that extended intervals may be acceptable to the majority of PWD if there was adequate evidence to support such a change [35]. We found that over a series of workshops the PPI volunteers became expert in the field and made important design decisions.

We believe that screening for referable DR at intervals based on individual risk could be both safe and cost-effective compared with annual screening. In this paper we report the key features of the design of an RCT designed to test this hypothesis.

Funding and ethics approval

For peer review only

The ISDR RCT is part of a larger programme of applied research funded by a £2.1m grant from the National Institute for Health Research (NIHR) Programme Grants for Applied Research (PGfAR) programme – reference RG-PG-1210-12016. SPH is the Chief Investigator for the 7 workstream programmes and DMB the Principal Investigator for the RCT. The RCT is supported by the Clinical Trials Research Centre (CTRC) in Liverpool, which provides information systems support, developed electronic case report files and manage the data. There are three trial oversight committees: a Trial Management Group, a Trial Steering Committee and the Independent Data and Safety Monitoring Committee. The composition and membership of the committees was approved by NIHR. The study protocol [36], patient information sheets and consent forms have received ethical approval from the National Research Ethics Service Committee North West – Preston (reference: 14/NW/0034). Recruitment to the trial was supported by research staff from Clinical Research Network (CRN) North West Coast, Liverpool Diabetic Eye Screening Programme (LDESP) staff and trained student volunteers.

Trial status

The trial opened to recruitment on the 12th November 2014. The first patient was randomised on the 19th November 2014. The trial was closed to enrolment on the 31 May 2016. The target of 4460 patients was reached. The Programme Grant completes on 30 April 2019.

Patient and Public Involvement

We have included members of our PPI Group in all stages of the design of the trial. Our PPI group consists of 7 individuals and has met at regular intervals throughout the lifetime of the trial. Individuals are active participants in the programme and trial steering committees,

For peer review only

programme investigators committee and trial management committee. Individuals were involved in the concept and development of the research questions, design of the trial and the grant application. One patient is a co-investigator and was closely involved in designing the intervention including reviewing the potential burden. The group was involved in the choice of risk factors for consideration for the RCE and secondary outcome measures. They developed a set of patient centred outcomes, most notably adding visual impairment and need for treatment which they viewed as particularly important to patients. They considered the chance of missing disease if screen intervals were extended and after several structured sessions settled on a 2.5% risk as an acceptable limit. They were not involved in actual recruitment to the trial but they visited the screening centres and observed recruitment and gave constructive comments which improved the process.

Regarding dissemination of results, these will be sent to the participants' General Practitioners. Participants have also been advised to let the researcher know if they would like a summary of the results themselves.

Participants, interventions and outcomes

Trial design

The ISDR RCT had 1:1 allocation to a personalised risk-based screening recall (6, 12 or 24 months) or annual screening (current routine care).

Objectives

The objective of the ISDR RCT is to evaluate the safety, acceptability and cost-effectiveness of personalised risk-based screening in a whole population setting. The main aim is to assess the relative safety of variable-interval screening as measured by attendance rate. We will test the hypothesis that the difference in attendance rates between the two pathways is within an acceptable equivalence margin.

For peer review only

Study setting

This single site trial is being conducted in all 7 screening clinics in the LDESP, which is part of the English National Diabetic Eye Screening Programme (NDESP).

Study population

People with diabetes registered with a GP whose postcode is in the city boundaries of Liverpool and attending for screening for DR were invited to enter the trial. The inclusion and exclusion criteria are given in Table 1.

**Inclusion Criteria**	**Exclusion Criteria**
adults, young people and children who are aged ≥12 years	under age 12 years
due to be offered an appointment for retinal screening during the recruitment period	
registered with a participating GP practice	are not registered with a participating GP practice
are included in the study data warehouse (have not opted out)	have opted out from the study data warehouse
have no retinopathy or have retinopathy and maculopathy less than the definition of screen positive diabetic retinopathy	have screen positive diabetic eye disease or significant other eye disease requiring referral to the HES
have gradeable digital retinal images in both eyes	are ineligible for screening for whatever reason, including having ungradable digital retinal images, which includes patients who have only

For peer review only

	one eye or an ungradable eye with no visual potential
give their informed consent for participation	do not give consent for participation in the RCT
are not involved in any trial investigating a treatment aiming at preventing or modifying the development of STDR	are involved in any trial investigating a treatment aiming at preventing or modifying the development of STDR

Table 1. Inclusion and exclusion criteria in the personalised variable-interval risk-based screening for diabetic retinopathy randomised controlled trial.

The Schematic of the ISDR RCT Study Design is given in Figure 1.

Data flows

Data flows are shown in Figure 2. Data on the participant's retinopathy status feeds automatically from the screening software (OptoMize) into the data warehouse and then to the randomisation and RCE programmes on a daily basis. The ISDR data warehouse automatically populates the majority of the fields in the baseline and follow-up electronic CRFs (OpenClinica), including data that allowed randomisation to occur.

Primary endpoint

The primary outcome is the attendance rate for follow-up screening in the two arms of the study. Non-attendance is defined as failure to attend any screening appointments within 90 days of the expected follow-up date, irrespective of how many appointments they had been sent.

Secondary endpoints

- Number of cases of STDR detected

For peer review only

- Retinopathy level at screening
- Maculopathy level at screening
- Number of false positive screening episodes
- Number of screening appointments
- Number of diabetes assessment clinic appointments
- Number of other eye appointments for DR
- Visual acuity (logMAR)
- New visual impairment (≥ +0.30 and ≥ +0.50 logMAR)
- New visual impairment (≥ +0.30 and ≥ +0.50 logMAR) which in the opinion of an experienced clinician is due to DR
- Number of missed appointments to screening
- Patient acceptability measures (using a questionnaire designed for the RCT)
- Quality-adjusted life years (QALYs) estimated using EQ-5D-5L and Health Utilities Index Mark 3 (HUI3)
- Cost per QALY

Intervention, assessments and procedures

The ISDR data warehouse (DW) is a relational database which stores both the data and how the data are related. The Liverpool GPs gave permission for their PWD to be directly contacted to give permission for the data held in primary care relating to their diabetes to be transferred to the DW. The DW was established to collate and dynamically link individual risk factor data from GP practices, STDR outcome data from the DR assessment clinic at St Pauls Eye Unit and the LDESP screening software (OptoMize). This central data repository

For peer review only

was used to develop the locally applicable risk calculation engine (RCE) described above using a continuous Markov model.

The RCE was developed using routinely collected primary and secondary care data from 11,806 PWD (46,525 screening episodes) in the LDESP. A detailed description of the RCE has already been published [32]. In brief, the final covariates for the RCE were selected using a 3-step decision process. A number of risk factors for developing STDR were identified in the published literature and in preliminary work in Liverpool. These potential covariates were reviewed by the clinical team and the PPI group and potential additional covariates were suggested. Potential covariates with a missingness rate of less than 20% in the data warehouse were identified. Then a statistical evaluation of the predictive value of each remaining covariate was undertaken. The covariates selected were disease state (current retinopathy levels in both eyes); age; duration of diabetes; glycated haemoglobin; systolic blood pressure; and total cholesterol. The results suggested that implementing personalised risk- based intervals would reduce the number of screen episodes by 30%.

Our PPI group defined the degree of risk (up to 2.5% risk of developing screen positive diabetic retinopathy) acceptable to people with diabetes, allowing assignment of individuals to screen intervals at the time points 6, 12 or 24 months. The choice of these intervals was based on a review of the available literature, our data and consensus between the PPI and research teams. Following each negative screening outcome, individuals are assigned to the longest recall period up to 24 months at which their risk estimation would not exceed the 2.5% threshold.

Consent

Individuals received a Patient Information Leaflet with all the details of the trial with their screening appointment reminder letter. On attending the screening clinic they were

For peer review only

approached by trained researchers to establish whether they wished to participate in the trial. If they expressed an interest the researcher took written informed consent and enrolled them into the trial. If an individual subsequently requested to be withdrawn from the trial they reverted to routine care. Reasons for, and level of, withdrawal were collected.

Randomisation

Randomisation could only be completed once the individual's retinal images had been graded, as it was only possible at this point to complete the inclusion and exclusion criteria. PWD who screened positive due to DR, other eye disease or ungradeable images were therefore consented but were not included in the trial.

Eligible PWD were randomly allocated to the personalised risk-based screening recall or the usual fixed annual interval. Participants were stratified by clinic and age (<16 and ≥16 years old). The screening clinics in Liverpool had different proportions of PWD in hard to reach groups, such as minority ethnic groups, attendance rates and social deprivation indices.

After randomisation the RCE was used to generate the individual's recall period in the personalised arm of the trial and the data was sent back to the screening software.

Study visits and assessments

At each follow-up visit, PWD randomised to the personalised risk-based screening arm have their risk re-calculated and the next recall interval determined accordingly.

Health economics

Table 2 shows the Trial Intervention diagram.

A health economics questionnaire, incorporating the health-related quality of life (QoL) questionnaires EQ-5D-5L [37] and Health Utilities Index Mark 3 (HUI3) [38] and a bespoke visit questionnaire [39], were completed by the first 868 eligible PWD enrolled into the

For peer review only

study (a minimum of 700 PWD was required to provide sufficient data). The QoL questionnaires are repeated at every subsequent follow-up visit for those PWD who completed it at baseline.

For peer review only

Table 2. Trial Intervention diagram

				Follow-Up Schedule							
Procedures	*Screening*	*Baseline*	*Randomisation*	*6 months***	*12 months***	*18 months***	*24 months***	*30 months***	*36 months***	*Study Completion*	*Premature Discontinuation*
Signed Consent Form	*X*										
Visual Acuity(LogMar)		*X*		*X*	*X*	*X*	*X*	*X*	*X*		
Visit Questionnaire		*X*									
QoL Questionaire (HUI3 and EQ5D)*		*X*		*X*	*X*	*X*	*X*	*X*	*X*		
Assessment of Eligibility Criteria	*X*	*X*	*X*								
Randomisation			*X*								
Digital Imaging		*X*		*X*	*X*	*X*	*X*	*X*	*X*		
Acceptability Questionnaire									*X*		

*The EQ-5D-5L, HUI3 data will be collected on around 100-200 eligible PWD and repeated at each visit for those individual PWD. Data will be stratified to balance equal numbers of PWD in each arm and representation from all screening sites.

**PWD are randomised to 2 arms. In one arm individuals attend at 12 month intervals. In the other arm individuals attend at 6, 12 or 24 monthly intervals.

Sample size and planned analyses

The primary aim is to assess the relative safety of personalised risk-based interval screening as measured by equivalence in attendance rates. The analysis will test the hypothesis that the difference in attendance rates at the first follow-up between the two pathways is within the acceptable range δ=0.05. If the bounds of the 90% confidence interval for the difference in attendance rate are found to be within the confidence limits $[-\delta, \delta]$ (i.e. [-0.05, 0.05]) the results would support equivalence.

The estimated minimum number of patients required is 3940. With an expected loss to follow-up rate of 6% per year due to death and other exclusion from screening (note that non-attendance is the primary outcome and therefore is not factored here) the target for recruitment (randomised into the trial) was 4460 patients (4460*0.94*0.94=3940).

A secondary aim is to investigate whether personalised screening can be considered as non-inferior in detection of STDR when compared to annual screening. The STDR detection rate predicted for the usual care pathway during the two years follow-up is approximately 6%, based on data from the LDESP. The sample size required to address the first question (n=4460 patients randomised with 3940 patients retained after 2 years from baseline) will permit us to test for non-inferiority in STDR detection with a maximum allowable reduction of 1.5% of the personalised care pathway compared to standard care, with 5% significance level and power between 60-65%.

We will undertake sub-group analyses to assess differences in attendance rates between the two arms for the three different retinopathy groups (the risk groups will be defined based on the individual baseline estimated risk of developing STDR). A logistic mixed-effects model that takes into account the patient's covariate information over time (including HbA1c, systolic blood pressure, total cholesterol, disease duration, retinopathy level, ethnicity, age, gender, smoking status, diabetes type), and screening clinic (clustered data) will be fitted with attendance (Yes/No) at the first screening visit as the outcome variable. The random effects of the model will account for the variability by screening clinic.

Using screening activity data stored in the ISDR DW and information collected from the visit questionnaire and EQ-5D-5L and HUI3 responses, we will estimate the cost per QALY within the study period associated with risk-based and annual screening from an NHS perspective and where possible incorporate a broader perspective. We will present bootstrapped incremental cost effectiveness ratios and cost effectiveness acceptability curves to characterise the uncertainty associated with our estimates.

We will apply sensitivity analyses to check the sensitivity of the results on the assumption that missing data are missing at random. Different scenarios for missing data mechanisms will be explored.

Conclusions

This paper describes the design of a RCT to evaluate the feasibility, safety, acceptability and cost-effectiveness of personalised variable-interval risk-based screening compared to fixed annual interval screening. Safety will be measured by the effect on attendance rates to screening, rates and severity of DR, visual outcomes and impact on general diabetes care. As far as we are aware this is the only study to investigate the impact of implementing personalised screening for diabetic retinopathy in an RCT.

For peer review only

Authors' contributions

DMB (Trial PI) and SPH (CI) conceived the trial questions and have been involved in all stages of the study design, and together with AEW (Trial Coordinator) and AW (ISDR Programme Manager) participated in writing the protocol, submission to the funding body and application to the ethics committee. SUH is a Senior Data Manager for the CTRC and DA is the information Systems Manager for the CTRC. Both contributed to writing the protocol and devised the electronic CRF system and data monitoring systems. MG-F is the lead statistician and co-investigator and CPC is the trial statistician. IMS is a leading UK statistician. All participated in writing the protocol and statistical aspects of the study. MMR is the data warehouse manager. MJ is the lead health economist and a co-investigator and CJS is the trial health economist. They designed the heath economic analysis. TM is a senior trials manager and a co-investigator. LH replaced AEW in the CTRC and has been involved in revising the protocol and preparing the manuscript with DMB and CJS. JK and JC are PPI members and contributed to the design of the RCE and the trial. All authors read and approved the final manuscript.

For peer review only

Legends

Figure 1: Schematic of the ISDR RCT trial design

Figure 2: Data Flows. In step 1 data for consented participants is requested from EMIS, passed to the Data Warehouse and cleaned prior to storage. In step 2 data is exchanged between the data warehouse and OptoMize (subjects whose risk needs to be calculated are sent to the data warehouse, subjects for whom their risk and therefore recall interval has been calculated is returned to OptoMize for appointment letter generation). In step 3 the participants risk is calculated (when all the covariates are available), if randomisation is

required they are then randomised. The data is then stored in the study database and the data warehouse. All processes in steps 1 and 2 involve identifiable data, the processes in step 3 all use pseudanonomised data for security reasons (the trial team with access to the trial database do not have a need to see raw identifiers). Step 1 occurs on a bi-monthy period, steps 2 & 3 occur on a daily basis. Under ideal conditions it takes three days for the data to pass through all parts of steps 1 and 2 – this is due to an airgap (manual transfer) at step 2 between the data warehouse and the NHS systems.

Table 1: Inclusion and exclusion criteria in the personalised variable-interval risk-based screening for diabetic retinopathy randomised controlled trial.

Table 2. Trial Intervention Diagram for the ISDR RCT

Competing interests

The authors report no competing interests

Funding statement

This paper presents independent research funded by the National Institute for Health Research (NIHR) under the Programme Grants for Applied Research programme (RP-PG-1210-12016). The views expressed are those of the authors and not necessarily those of the NHS, the NIHR or the Department of Health.

Data sharing statement

The formal protocol is published at 2014 DOI: 10.6084/m9.figshare.5053336.v1

Trial sponsors

University of Liverpool Research Support Office,

1st floor Duncan Building,

Daulby Street,

For peer review only

Liverpool L69 3GA.

Tel: 0151 706 3702

Research and Innovation Department

Royal Liverpool and Broadgreen University Hospitals Trust

Prescot Street

Liverpool

L7 8XP

0151 706 2000

University of Liverpool: UoL000994

Research and Innovation Department

Royal Liverpool and Broadgreen University Hospitals Trust

Prescot Street

Liverpool

L7 8XP

0151 706 2000

Trust RD&I: 4660

ISDR Study Group

Simon P Harding (Study Group Chair), Deborah M Broadbent (Trial PI), Anthony C Fisher, Mark Gabbay, Marta García-Fiñana, Marilyn James, Tracy Moitt, John R Roberts, Daniel Seddon, Irene M Stratton, Paula Williamson, Duncan Appelbe, Andy Ovens, Lola Howard, Susan U Howlin, Ayesh Alshukri, Abigail E Williams, Christopher P Cheyne, Antonio Eleuteri, Christopher Grierson, Mehrdad Mobayen-Rahni, Christopher J Sampson, David Szmyt, Clare Thetford, Maria Pilar Vazquez Arango, Amu Wang, Helen Cooper, John Collins, John Kelly, Nathalie Massat, Gideon Smith, Vineeth Kumar, Chris Rogers, Julia West, Naveed Younis,

For peer review only

Alison Rowlands, Peter Lees, Sandra Lees, Emily Doncaster, Betty Williams, Ticiana Criddle, Stephanie Perrett.

Acknowledgments

We would like to acknowledge the outstanding attention to detail from the ISDR administrative team and the CTRC. We would like to thank the North West Coast CRN and our other trained researchers for their invaluable help in recruiting to target. We would also like to thank the LDESP for their support and assistance. We would particularly like to thank our PPI members for their invaluable advice and enthusiastic support for the study.

Christopher J Sampson is currently affiliated to the Office of Health Economics, Southside, 7th Floor, 105 Victoria Street, London SW1E 6QT

References

1. PHE bulletin 2016 https://www.gov.uk/government/uploads/system/uploads/attachment_data/file/556910/PHE_Bulletin_September_2016.pdf Last accessed 03.01.18.

2. Klein R, Klein BE, Moss SE, Davis MD, DeMets DL. The Wisconsin epidemiologic study of diabetic retinopathy. II. Prevalence and risk of diabetic retinopathy when age at diagnosis is less than 30 years. *Arch Ophthalmol* 1984, 102:520-526.

3. Klein R, Klein BE, Moss SE, Davis MD, DeMets DL. The Wisconsin epidemiologic study of diabetic retinopathy. III. Prevalence and risk of diabetic retinopathy when age at diagnosis is 30 or more years. *Arch Ophthalmol* 1984, 102:527-532.

4. Liew, G, Michaelides, M, Bunce, C. A comparison of the causes of blindness certifications in England and Wales in working age adults (16 to 64 years), 1999 to 2000 with 2009 to 2010. *BMJ Open* 2014, 12 February 2014.

For peer review only

5. Javitt JC, Aiello LP: Cost-Effectiveness of Detecting and Treating Diabetic Retinopathy. *Annals of Internal Medicine* 1996, 124:164-169.

6. Jones S, Edwards RT. Diabetic retinopathy screening: a systematic review of the economic evidence. *Diabet Med* 2010, 27(3):249–56. DOI: 10.1111/j.1464-5491.2009.02870.x

7. James M, Turner DA, Broadbent DM, Vora J, Harding SP. Cost effectiveness analysis of screening for sight threatening diabetic eye disease. *BMJ* 2000, 320(7250):1627–31.

8. UK National Screening Committee. The UK NSC recommendation on Diabetic Retinopathy screening in adults. January 2016. Available at http://legacy.screening.nhs.uk/diabeticretinopathy. Last accessed 03.01.18.

9. Scanlon PH, Aldington, SJ, Leal J, Luengo-Fernandez R, Oke J, Sivaprasad S et al. Development of a cost-effectiveness model for optimisation of the screening interval in diabetic retinopathy screening. *Health Technol Assess* 2015;19:1-116.

10. Taylor-Phillips S, Mistry H, Leslie R, Todkill D, Tsertd vadze A, Connock M et al. Extending the diabetic retinopathy screening interval beyond 1 year: systematic review. *Br J Ophthalmol* 2016;100:105-114.

11. Leese GP, Stratton IM, Land M, Bachmann MO, Jones C, Scanlon P et al. Progressio of diabetes retinal status within community screening programs and potential implications for screening intervals. *Diabetes Care* 2015;38:488-494.

12. Davies R, Sullivan P, Canning C. Simulation of diabetic eye disease to compare screening policies. *Br J Ophthalmol* 1996, 80:945-950.

13. Younis N, Broadbent DM, Vora JP, Harding SP. Incidence of sight-threatening retinopathy in patients with Type 2 diabetes in the Liverpool Diabetic Eye Study: a cohort study. *Lancet* 2003, 361:195-200.

For peer review only

14. Younis N, Broadbent DM, Harding SP, Vora JP. Incidence of sight-threatening retinopathy in Type 1 diabetes in a systematic screening programme. *Diabet Med* 2003, 20:758-765.

15. Stefansson E, Bek T, Porta M, Larsen N, Kristinsson JK, Agardh E. Screening and prevention of diabetic blindness. *Acta Ophthalmol Scand* 2000;78:374-385.

16. Agardh E, Tabayat-Khani P. Adopting 3-year screening intervals for sight-threatening retinal vascular lesions in Type 2 diabetic subjects without retinopathy. *Diab Care* 2011;34:1-2.

17. Stratton IM, Aldington SJ, Taylor DJ, Adler AI, Scanlon PH. A simple risk stratification for time to development of sight-threatening diabetic retinopathy. *Diabetes Care* 2013 Mar; 36(3):580-5. DOI: 10.2337/dc12-0625.

18. Scanlon PH, Stratton IM, Histed M, Chave SJ, Aldington SJ. The influence of background diabetic retinopathy in the second eye on rates of progression of diabetic retinopathy between 2005 and 2010. *Acta Ophthalmol* 2013 Aug; 91(5):e335-9. DOI: 10.1111/aos.12074.

19. Taylor-Phillips S, Mistry H, Leslie R et al. Extending the diabetic retinopathy screening interval beyond 1 year: systematic review. http://dx.doi.org/10.1136/bjophthalmol-2014-305938.

20. The Diabetes Control and Complications Trial Research Group. The effect of intensive treatment of diabetes on the development and progression of long-term complications in insulin-dependent diabetes mellitus. *N Engl J Med* 1993, 329:977-986.

21. Davis MD, Fisher MR, Gangnon RE, Barton F, Aiello LM, Chew EY, Ferris FL, 3rd, Knatterud GL. Risk factors for high-risk proliferative diabetic retinopathy and severe visual

For peer review only

loss: Early Treatment Diabetic Retinopathy Study Report #18. *Invest Ophthalmol Vis Sci* 1998, 39:233-252.

22. Marshall G, Garg SK, Jackson WE, Holmes DL, Chase HP. Factors influencing the onset and progression of diabetic retinopathy in subjects with insulin-dependent diabetes mellitus. *Ophthalmology* 1993, 100:1133-1139.

23. Stratton IM, Kohner EM, Aldington SJ, Turner RC, Holman RR, Manley SE, Matthews DR: UKPDS 50. Risk factors for incidence and progression of retinopathy in Type II diabetes over 6 years from diagnosis. *Diabetologia* 2001, 44:156-163.

24. Agardh CD, Agardh E, Torffvit O. The association between retinopathy, nephropathy, cardiovascular disease and long-term metabolic control in Type 1 diabetes mellitus: a 5 year follow-up study of 442 adult patients in routine care. *Diabetes Res Clin Pract* 1997, 35:113-121.

25. Vijan S, Hofer TP, Hayward RA. Estimated Benefits of Glycemic Control in Microvascular Complications in Type 2 Diabetes. *Annals of Internal Medicine* 1997, 127:788-795.

26. UK Prospective Diabetes Study Group. Tight blood pressure control and risk of macrovascular and microvascular complications in Type 2 diabetes: UKPDS 38. . *BMJ* 1998, 317:703-713.

27. Gallego PH, Craig ME, Hing S, Donaghue KC. Role of blood pressure in development of early retinopathy in adolescents with Type 1 diabetes: prospective cohort study. *BMJ* 2008, 337:a918.

28. Chaturvedi N, Sjolie AK, Stephenson JM, Abrahamian H, Keipes M, Castellarin A, Rogulja-Pepeonik Z, Fuller JH. Effect of lisinopril on progression of retinopathy in

For peer review only

normotensive people with Type 1 diabetes. The EUCLID Study Group. EURODIAB Controlled Trial of Lisinopril in Insulin-Dependent Diabetes Mellitus. *Lancet* 1998, 351:28-31.

29. Keech A, Simes RJ, Barter P, Best J, Scott R, Taskinen MR, Forder P, Pillai A, Davis T, Glasziou P, et al. Effects of long-term fenofibrate therapy on cardiovascular events in 9795 people with Type 2 diabetes mellitus (the FIELD study): randomised controlled trial. *Lancet* 2005, 366:1849-1861.

30. Klein R, Klein BEK, Moss SE, Cruickshanks KJ. The Wisconsin epidemiologic study of diabetic retinopathy: XVII: The 14-year incidence and progression of diabetic retinopathy and associated risk factors in Type 1 diabetes. *Ophthalmology* 1998, 105:1801-1815.

31. Sampson CJ, James M, Broadbent DM, Harding SP. Stratifying the NHS Diabetic Eye Screening Programme: into the unknown? *Diabet Med* 2016, 33(12): 1612–4. DOI: 10.1111/dme.13192.

32. Eleuteri A, Fisher AC, Broadbent DM, García-Fiñana M, Cheyne CP, Wang A, Stratton IM, Gabbay M, Seddon D, Harding SP for the ISDR Study Group. Individualised variable interval risk-based screening for sight threatening diabetic retinopathy – the Liverpool Risk Calculation Engine. *Diabetologia* 2017, 60(11): 2174-2182.

33. Aspelund T, Porisdottir O, Olafsdottir E, Gudmundsdottir A, Einarsdottir AB, Mehlsen J, Einarsson S, Palsson O, Bek T, Stefansson E. Individual risk assessment and information technology to optimise screening frequency for diabetic retinopathy. *Diabetologia* 2011, DOI: 10.1007/s00125-011-2257-7.

34. van der Heijden AA, Walraven I, van't Riet E et al. Validation of a model to estimate personalised screening frequency to monitor diabetic retinopathy. *Diabetologia* 2014, 57(7):1332-1338.

For peer review only

35. Yeo ST, Edwards RT, Luzio SD, Charles JM, Thomas RL, Peters JM, et al. Diabetic retinopathy screening: perspectives of people with diabetes, screening intervals and costs of attending screening. *Diabet Med* 2012, 29(7):878–85. DOI: 10.1111/j.1464-5491.2012.03637.x

36. Broadbent DM, Bennett A, Moitt T, Howlin S, James M, Sampson C, et al. Introducing personalised risk based intervals in screening for diabetic retinopathy: development, implementation and assessment of safety, cost-effectiveness and patient experience. Workstream E: Randomised trial comparing standard and test screening intervals protocols. 2014 DOI: 10.6084/m9.figshare.5053336.v1.

37. Herdman M, Gudex C, Lloyd A, Janssen M, Kind P, Parkin D, et al. Development and preliminary testing of the new five-level version of EQ-5D (EQ-5D-5L). *Qual Life Res* 2011, 20(10):1727–36. DOI: 10.1007/s11136-011-9903-x

38. Feeny D, Furlong W, Boyle M, Torrance GW. Multi-attribute health status classification systems. Health Utilities Index. *Pharmacoeconomics* 1995, 7(6):490–502. DOI: 10.2165/00019053-199507060-00004.

39. James M, Sampson C. ISDR Visit Questionnaire. Database of Instruments for Resource Use Measurement. 2015. DOI: Available from: http://dirum.org/instruments/details/90 DOI: 10.6084/m9.figshare.1301022.v1.

I, the Submitting Author has the right to grant and does grant on behalf of all authors of the Work (as defined in the below author licence), an exclusive licence and/or a non-exclusive licence for contributions from authors who are: i) UK Crown employees; ii) where BMJ has agreed a CC-BY licence shall apply, and/or iii) in accordance with the terms applicable for US Federal Government officers or employees acting as part of their official duties; on a worldwide, perpetual, irrevocable, royalty-free basis to BMJ Publishing Group Ltd ("BMJ") its licensees and where the relevant Journal is co-owned by BMJ to the co-owners of the Journal, to publish the Work in BMJ Open and any other BMJ products and to exploit all rights, as set out in our licence.

For peer review only

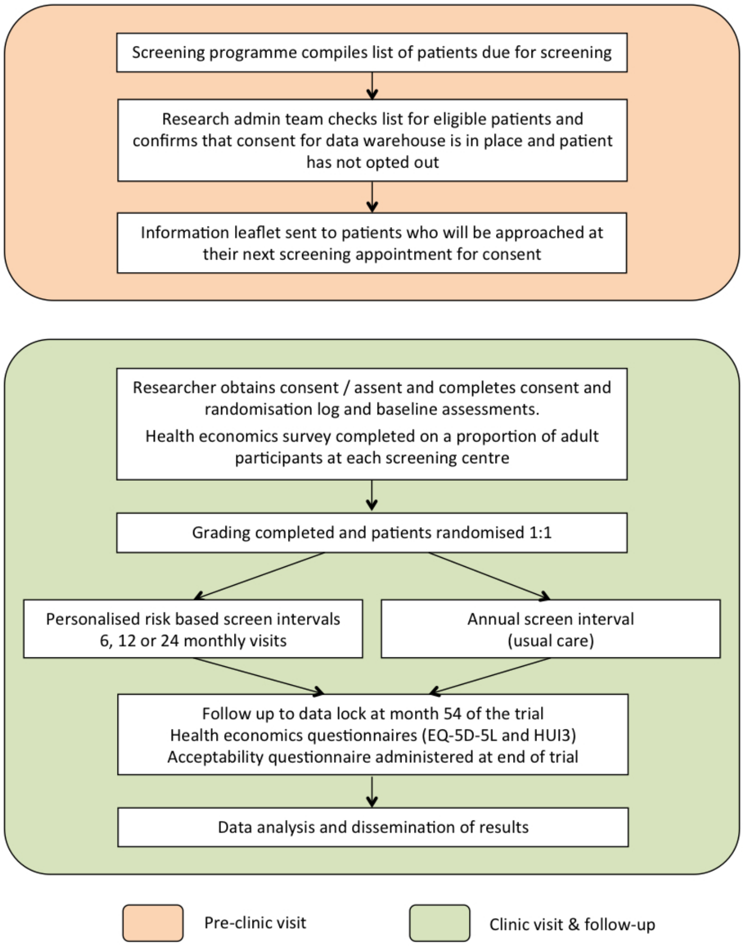

Figure 1: Schematic of the ISDR RCT trial design

352x470mm (72 x 72 DPI)

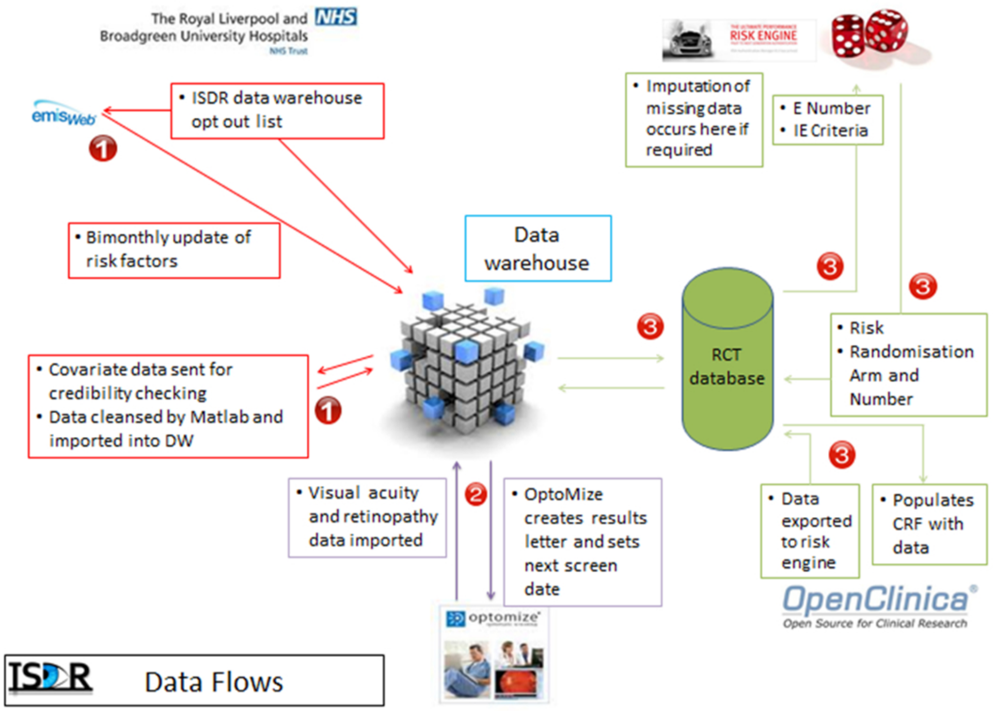

Figure 2: Data Flows. In step 1 data for consented participants is requested from EMIS, passed to the Data Warehouse and cleaned prior to storage. In step 2 data is exchanged between the data warehouse and OptoMize (subjects whose risk needs to be calculated are sent to the data warehouse, subjects for whom their risk and therefore recall interval has been calculated is returned to OptoMize for appointment letter generation). In step 3 the participants risk is calculated (when all the covariates are available), if randomisation is required they are then randomised. The data is then stored in the study database and the data warehouse. All processes in steps 1 and 2 involve identifiable data, the processes in step 3 all use pseudanonomised data for security reasons (the trial team with access to the trial database do not have a need to see raw identifiers). Step 1 occurs on a bi-monthy period, steps 2 & 3 occur on a daily basis. Under ideal conditions it takes three days for the data to pass through all parts of steps 1 and 2 – this is due to an airgap (manual transfer) at step 2 between the data warehouse and the NHS systems.

90x90mm (300 x 300 DPI)

STANDARD PROTOCOL ITEMS: RECOMMENDATIONS FOR INTERVENTIONAL TRIALS

SPIRIT 2013 Checklist: Recommended items to address in a clinical trial protocol and related documents*

Section/item	Item No	Description	Addressed on page number
**Administrative information**			
Title	1	Descriptive title identifying the study design, population, interventions, and, if applicable, trial acronym	___3_________
Trial registration	2a	Trial identifier and registry name. If not yet registered, name of intended registry	___6_________
	2b	All items from the World Health Organization Trial Registration Data Set	__NA**________
Protocol version	3	Date and version identifier	__NA**________
Funding	4	Sources and types of financial, material, and other support	___11________
Roles and responsibilities	5a	Names, affiliations, and roles of protocol contributors	____3________
	5b	Name and contact information for the trial sponsor	____22/23___
	5c	Role of study sponsor and funders, if any, in study design; collection, management, analysis, and interpretation of data; writing of the report; and the decision to submit the report for publication, including whether they will have ultimate authority over any of these activities	____22_______
	5d	Composition, roles, and responsibilities of the coordinating centre, steering committee, endpoint adjudication committee, data management team, and other individuals or groups overseeing the trial, if applicable (see Item 21a for data monitoring committee)	____11________

For peer review only - http://bmjopen.bmj.com/site/about/guidelines.xhtml

Introduction

Background and rationale	6a	Description of research question and justification for undertaking the trial, including summary of relevant studies (published and unpublished) examining benefits and harms for each intervention	____7-11_____
	6b	Explanation for choice of comparators	____7-11_____
Objectives	7	Specific objectives or hypotheses	_____13______
Trial design	8	Description of trial design including type of trial (eg, parallel group, crossover, factorial, single group), allocation ratio, and framework (eg, superiority, equivalence, noninferiority, exploratory)	_____13______

Methods: Participants, interventions, and outcomes

Study setting	9	Description of study settings (eg, community clinic, academic hospital) and list of countries where data will be collected. Reference to where list of study sites can be obtained	_____13______
Eligibility criteria	10	Inclusion and exclusion criteria for participants. If applicable, eligibility criteria for study centres and individuals who will perform the interventions (eg, surgeons, psychotherapists)	_Table 1_____
Interventions	11a	Interventions for each group with sufficient detail to allow replication, including how and when they will be administered	____13,16/17__
	11b	Criteria for discontinuing or modifying allocated interventions for a given trial participant (eg, drug dose change in response to harms, participant request, or improving/worsening disease)	____17 _____
	11c	Strategies to improve adherence to intervention protocols, and any procedures for monitoring adherence (eg, drug tablet return, laboratory tests)	___NA_______
	11d	Relevant concomitant care and interventions that are permitted or prohibited during the trial	___NA_______
Outcomes	12	Primary, secondary, and other outcomes, including the specific measurement variable (eg, systolic blood pressure), analysis metric (eg, change from baseline, final value, time to event), method of aggregation (eg, median, proportion), and time point for each outcome. Explanation of the clinical relevance of chosen efficacy and harm outcomes is strongly recommended	____15_______
Participant timeline	13	Time schedule of enrolment, interventions (including any run-ins and washouts), assessments, and visits for participants. A schematic diagram is highly recommended (see Figure)	___Fig 1_____

For peer review only - http://bmjopen.bmj.com/site/about/guidelines.xhtml

Sample size	14	Estimated number of participants needed to achieve study objectives and how it was determined, including clinical and statistical assumptions supporting any sample size calculations	____19-20____
Recruitment	15	Strategies for achieving adequate participant enrolment to reach target sample size	_____achieved

Methods: Assignment of interventions (for controlled trials)

Allocation:

Sequence generation	16a	Method of generating the allocation sequence (eg, computer-generated random numbers), and list of any factors for stratification. To reduce predictability of a random sequence, details of any planned restriction (eg, blocking) should be provided in a separate document that is unavailable to those who enrol participants or assign interventions	_____17____
Allocation concealment mechanism	16b	Mechanism of implementing the allocation sequence (eg, central telephone; sequentially numbered, opaque, sealed envelopes), describing any steps to conceal the sequence until interventions are assigned	_____17____
Implementation	16c	Who will generate the allocation sequence, who will enrol participants, and who will assign participants to interventions	_____17____
Blinding (masking)	17a	Who will be blinded after assignment to interventions (eg, trial participants, care providers, outcome assessors, data analysts), and how	____NA______
	17b	If blinded, circumstances under which unblinding is permissible, and procedure for revealing a participant's allocated intervention during the trial	____NA______

Methods: Data collection, management, and analysis

Data collection methods	18a	Plans for assessment and collection of outcome, baseline, and other trial data, including any related processes to promote data quality (eg, duplicate measurements, training of assessors) and a description of study instruments (eg, questionnaires, laboratory tests) along with their reliability and validity, if known. Reference to where data collection forms can be found, if not in the protocol	______11_____
	18b	Plans to promote participant retention and complete follow-up, including list of any outcome data to be collected for participants who discontinue or deviate from intervention protocols	____NA**_____

Data management	19	Plans for data entry, coding, security, and storage, including any related processes to promote data quality (eg, double data entry; range checks for data values). Reference to where details of data management procedures can be found, if not in the protocol	_____11______
Statistical methods	20a	Statistical methods for analysing primary and secondary outcomes. Reference to where other details of the statistical analysis plan can be found, if not in the protocol	______19-20___
	20b	Methods for any additional analyses (eg, subgroup and adjusted analyses)	______19-20___
	20c	Definition of analysis population relating to protocol non-adherence (eg, as randomised analysis), and any statistical methods to handle missing data (eg, multiple imputation)	______19-20___
**Methods: Monitoring**			
Data monitoring	21a	Composition of data monitoring committee (DMC); summary of its role and reporting structure; statement of whether it is independent from the sponsor and competing interests; and reference to where further details about its charter can be found, if not in the protocol. Alternatively, an explanation of why a DMC is not needed	______11**____
	21b	Description of any interim analyses and stopping guidelines, including who will have access to these interim results and make the final decision to terminate the trial	___ NA**_____
Harms	22	Plans for collecting, assessing, reporting, and managing solicited and spontaneously reported adverse events and other unintended effects of trial interventions or trial conduct	____NA**______
Auditing	23	Frequency and procedures for auditing trial conduct, if any, and whether the process will be independent from investigators and the sponsor	_____11______
**Ethics and dissemination**			
Research ethics approval	24	Plans for seeking research ethics committee/institutional review board (REC/IRB) approval	_____11_____
Protocol amendments	25	Plans for communicating important protocol modifications (eg, changes to eligibility criteria, outcomes, analyses) to relevant parties (eg, investigators, REC/IRBs, trial participants, trial registries, journals, regulators)	_____NA_____

For peer review only - http://bmjopen.bmj.com/site/about/guidelines.xhtml

Consent or assent	26a	Who will obtain informed consent or assent from potential trial participants or authorised surrogates, and how (see Item 32)	_____17_______
	26b	Additional consent provisions for collection and use of participant data and biological specimens in ancillary studies, if applicable	_____NA_______
Confidentiality	27	How personal information about potential and enrolled participants will be collected, shared, and maintained in order to protect confidentiality before, during, and after the trial	_____11**_______
Declaration of interests	28	Financial and other competing interests for principal investigators for the overall trial and each study site	_____22______
Access to data	29	Statement of who will have access to the final trial dataset, and disclosure of contractual agreements that limit such access for investigators	______NA_______
Ancillary and post-trial care	30	Provisions, if any, for ancillary and post-trial care, and for compensation to those who suffer harm from trial participation	______NA_____
Dissemination policy	31a	Plans for investigators and sponsor to communicate trial results to participants, healthcare professionals, the public, and other relevant groups (eg, via publication, reporting in results databases, or other data sharing arrangements), including any publication restrictions	______13______
	31b	Authorship eligibility guidelines and any intended use of professional writers	____ NA**______
	31c	Plans, if any, for granting public access to the full protocol, participant-level dataset, and statistical code	______NA______
**Appendices**			
Informed consent materials	32	Model consent form and other related documentation given to participants and authorised surrogates	___NA________
Biological specimens	33	Plans for collection, laboratory evaluation, and storage of biological specimens for genetic or molecular analysis in the current trial and for future use in ancillary studies, if applicable	___NA________

*It is strongly recommended that this checklist be read in conjunction with the SPIRIT 2013 Explanation & Elaboration for important clarification on the items. Amendments to the protocol should be tracked and dated. The SPIRIT checklist is copyrighted by the SPIRIT Group under the Creative Commons "Attribution-NonCommercial-NoDerivs 3.0 Unported" license.

For peer review only - http://bmjopen.bmj.com/site/about/guidelines.xhtml

** This is a Rationale paper rather than a published protocol. Where asterisked these details are available in the Protocol at 2014 DOI: 10.6084/m9.figshare.5053336.v1

For peer review only

For peer review only - http://bmjopen.bmj.com/site/about/guidelines.xhtml
